# Receptor tyrosine kinase ROR1 ameliorates Aβ_1–42_ induced cytoskeletal instability and is regulated by the miR146a-NEAT1 nexus in Alzheimer’s disease

**DOI:** 10.1038/s41598-021-98882-0

**Published:** 2021-09-28

**Authors:** Kaushik Chanda, Nihar Ranjan Jana, Debashis Mukhopadhyay

**Affiliations:** 1 Biophysics and Structural Genomics Division, Saha Institute of Nuclear Physics, HBNI, Block-AF, Sector 1, Bidhannagar, Kolkata, WB 700 064 India; 2 Cellular and Molecular Neuroscience Laboratory, National Brain Research Centre, Manesar, Gurgaon, 122 050 India; 3 Present Address: School of Bioscience, Indian Institute of Technology, Kharagpur, India

**Keywords:** Alzheimer's disease, Long non-coding RNAs, miRNAs, Mechanisms of disease

## Abstract

Alzheimer’s disease (AD) involves severe cytoskeletal degradation and microtubule disruption. Here, we studied the altered dynamics of ROR1, a Receptor Tyrosine Kinase (RTK), and how it could counter these abnormalities. We found that in an Aβ_1–42_ treated cell model of AD, ROR1 was significantly decreased. Over expressed ROR1 led to the abrogation of cytoskeletal protein degradation, even in the presence of Aβ_1–42,_ preserved the actin network, altered actin dynamics and promoted neuritogenesis. Bioinformatically predicted miRNAs hsa-miR-146a and 34a were strongly up regulated in the cell model and their over expression repressed ROR1. LncRNA NEAT1, an interactor of these miRNAs, was elevated in mice AD brain and cell model concordantly. RNA Immunoprecipitation confirmed a physical interaction between the miRNAs and NEAT1. Intuitively, a transient knock down of NEAT1 increased their levels. To our knowledge, this is the first instance which implicates ROR1 in AD and proposes its role in preserving the cytoskeleton. The signalling modalities are uniquely analyzed from the regulatory perspectives with miR-146a and miR-34a repressing ROR1 and in turn getting regulated by NEAT1.

## Introduction

Alzheimer’s disease (AD) is characterised by a gradual and continuous loss of memory retention and cognition. The cellular abnormalities evident in Alzheimer’s disease pathology comprise of, among other things, a gross dysregulation of the cytoskeleton network^1^. While, there are two divergent ideas viz. β-amyloid (Aβ) plaques^2–4^ and neurofibrillary tangles (NFTs) as to the causality of the same, the end result is the loss of the neuronal cytoskeleton integrity, both spatially and temporally. Several studies have implicated the specific role of Aβ in disrupting the normal cytoskeleton by affecting the Amyloid Precursor Protein (APP) trafficking, altering the filamentous actin ratio and changing the epigenetic signatures of microtubule proteins^5,6^. In recent years, the direct effect of Aβ on regulating the actin dynamics via the protein Cofilin has been established^7–9^.

Receptor Tyrosine Kinases (RTKs) are a class of cell surface membrane proteins capable of autophosphorylation on cues of extracellular ligands and transmitting that signal downstream. RTKs have been shown to govern critical aspects of neuronal development and physiology like axonal growth cone guidance, synaptic signal transduction, cell to cell communication, among others^10–12^. In the last decade, RTK-like orphan receptor (Ror) proteins have come into prominence governing crucial physiological and developmental aspects like motility, polarity determination, Wnt signalling modulation, skeletal and cardiac system development^13,14^. The Ror family comprises of ROR1, ROR2 and Ryk. Of these, ROR1 and ROR2 are specifically implicated in the process of neurite extension and neurogenesis—two events critical to establish neuronal network^15–17^. Transient knock down led to shorter neuritic processes whereas, their over expression led to formation of highly branched processes. MAP1B and MAP2, two critical microtubule associated proteins, were found to change significantly upon Ror intervention^18^.

Additionally in the recent past, several miRNAs have been identified to be key modulators of cellular cytoskeleton circuitry. miR-142 affects the critical proteins required for the cytoskeleton dynamics in mature megakaryocytes^19^ targeting several actin cytoskeleton-associated proteins, like Cofilin-2 (Cfl2), Glucocorticoid receptor DNA binding factor 1 (Grlf1), Biorientation of chromosomes in cell division 1 (Bod1), Integrin alpha V (ItgaV) among others. miR-34-5p is found to regulate the expression of cytoskeleton genes during early insect development and segmentation^20^. Increased miR-155 levels in human endothelial cells affects the morphology and filamentous (F)-actin organization and the molecule targets EC cytoskeleton components RhoA and myosin light chain kinase (MYLK)^21^. Several other miRNAs also target key proteins in AD, like miR-106a, miR-106b, miR-520c, miR-101 and miR-153 directly target Amyloid Precursor Protein and control Aβ levels; miR-29a/b/c, miR-107, miR-195 and miR-124 targets BACE1, thereby regulating the intramembrane cleavage of APP; miR-132 targets Tau; miR-34a targets TREM2 and miR-146a targets a critical component of actin modulator, ROCK1^22^. It is interesting to note that miR-146a, implicated in AD, also affects the actin dynamics by targeting RhoA^23^. Similarly, miR-34a which regulates RhoA/Rac1 levels^24^ has a strong correlation to AD.

Besides Aβ oligomers, RTK signalling and miRNA mediated regulations, an emerging subset of long non-coding RNAs (lncRNAs) have been implicated in governing the cytoskeleton. lncRNA Down-regulated in hepatocellular carcinoma (Dreh) regulates vimentin, changing the cytoskeleton structure and cell morphology in cancer cells^25,26^. LINC00152 alters the F-actin dynamics by perturbing the levels of GOLPH3^27,28^. GAS5 suppresses glioma proliferation, migration and invasion by sponging miR-222. MiR-222 in turn is implicated in cofilin dephosphorylation^29,30^. UCA1 regulates hsa-miR-145–ZEB1/2–FSCN1 pathway in bladder cancer^31^. Apart from directly exerting effects on the cytoskeleton, several lncRNAs act indirectly via the small GTPases Rho, Rac1 and Cdc42. LncRNA LERFS (lowly expressed in rheumatoid fibroblast-like synoviocytes) quenches RhoA, Rac1, and Cdc42 in fibroblast-like synoviocytes^32^. PCGEM1 (prostate cancer gene expression marker 1) and TBILA (TGFβ-induced lncRNA) activates RhoA^33,34^. LncRNA TUNAR (neural differentiation-associated RNA) sponges miR-200a which suppresses the expression of Rac1^35^. The lncRNA H19 and SNHG15 (small nucleolar RNA host gene 15) upregulate Cdc42 expression by acting as buffers for associated miRNAs^36,37^. In recent times Nuclear Enriched Abundant Transcript 1 (NEAT1) has come to the forefront of research. Originally discovered as a virus inducible RNA^38^, NEAT1 is specifically located in the nuclear substructures called paraspeckles, and is essential for their formation and maintenance^39,40^. Of late, NEAT1 has been implicated in several types of cancers, including, but not limited to ovarian, prostrate, non-small lung, breast and hepatocellular carcinoma^41–45^. Apart from diverse carcinogenesis, NEAT1 has also been studied in several neurodegenerative scenarios namely Fronto-temporal dementia, Amyotrophic lateral sclerosis, Huntington’s disease and Parkinson’s disease^46–49^. Although the exact mechanism of action is unknown, research suggests that interaction of NEAT1 with associated miRNAs could be one paradigm which governs disease pathology. Such regulatory networks have been reported involving NEAT1 in Alzheimer’s disease as well^50,51^, but the knowledge is rudimentary.

At this backdrop, where it is evident that diverse molecules through their crosstalk could actually influence the cytoskeletal integrity in AD like degeneration, we would like to focus on the RTK ROR1. We postulate that its deregulation could potentially affect the associated components systemically, ending at the level of lncRNAs. The goals of the present study are to (1) access the levels of ROR1 in an AD cell model, (2) identify and validate miRNA repressors of ROR1 and their status in AD, (3) identify and validate the lncRNAs associated with said miRNAs, and (4) functionally link the RTK-miRNA-lncRNA regulatory network in AD.

## Results

### ROR1 and key cytoskeletal proteins are deregulated in Aβ_1–42_ treated cell model compromising the cytoskeletal architecture

**Figure 1:**
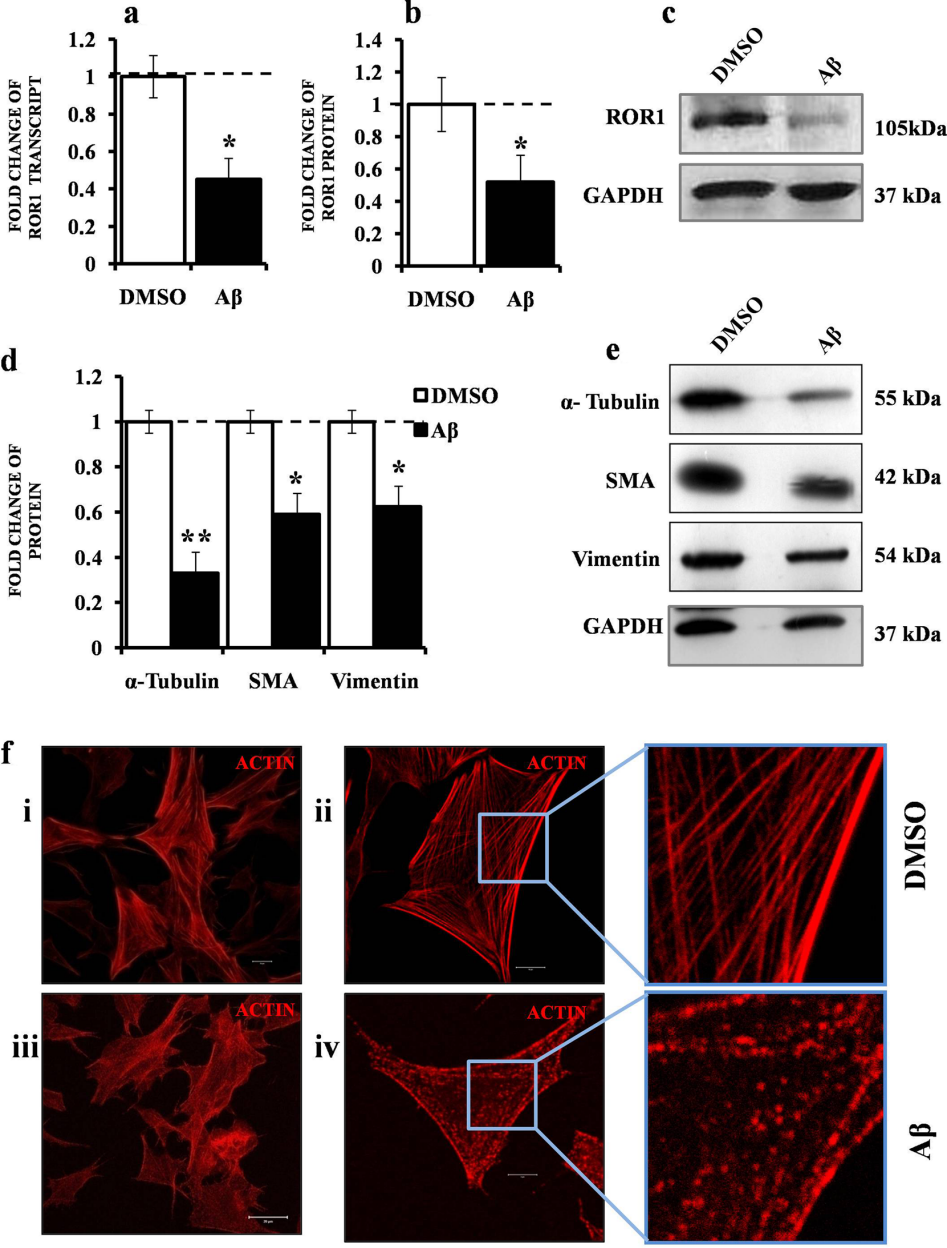
Deregulation of ROR1, key signalling proteins and actin cytoskeleton in Aβ_1–42_ treated cell model. (**a**) Graph depicting three (n = 3) independent biological replicates quantifying levels of ROR1 by qRT-PCR in SHSY-5Y cells treated with 1 µM Aβ_1–42_ or treated with only DMSO. (**b**) Graph depicting the mean value of optical density of the ROR1 bands, normalized against GAPDH. (**c**) Western blot (n = 3) showing the ROR1 and GAPDH levels in Aβ_1–42_ treated cell model. (**d**) Graph depicting the mean value of optical density of the α-Tubulin, SMA and Vimentin bands, normalized against GAPDH. (**e**) Western blot (n = 3) showing the α-Tubulin, SMA, Vimentin and GAPDH levels in Aβ_1–42_ treated cell model (**f**). Confocal microscopy images of phalloidin-561 (actin) stained SHSY-5Y cells; DMSO treated (Panel i. 1X zoom), DMSO treated (Panel ii. and inset. 3X zoom), Scale bars, 5 μm; Aβ_1–42_ treated (Panel iii. 1X zoom), Aβ_1–42_ treated (Panel iv. and inset. 3X zoom), Scale bars, 5 μm; For each confocal experiment, images of at least 30 cells (or cell fields) were captured and the experiments were repeated thrice (n = 3). Error bars indicate ± SD. Significance level between different experimental pairs is shown (NS, not significant; *p < 0.05; **p < 0.01; ***p < 0.001).

To begin with, we looked at the deregulated levels of ROR1, both at the transcript and protein levels in SHSY-5Y cells treated with Aβ_1–42_ and compared with DMSO control (considered as 1). In order to check if Aβ_1–42_ elicited cell cytotoxicity and death, we performed cell viability assays. Both MTT and Trypan Blue experiments showed that the Aβ_1–42_ concentration used here was sufficient to reduce the SHSY-5Y population by nearly 50% (Supplementary Fig. 1). Subsequently, both mRNA (fold change 0.43 Fig. 1a) and protein levels (fold change 0.37 Fig. 1b,c) showed that treatment with Aβ_1–42_ elicited downregulation of ROR1. Owing to ROR1’s association with cytoskeleton, we wanted to see if our cell model showed deregulation of cytoskeletal representative proteins, namely α-Tubulin (microtubule), Smooth Muscle Actin (SMA) (intermediate filament) and Vimentin (microfilament). On exposure to Aβ_1–42_, the levels of α-Tubulin (fold change 0.33), SMA (fold change 0.59) and Vimentin (fold change 0.62) decreased significantly (Fig. 1d,e). The same treatment was also sufficient to show visible phenotypic changes in the actin network of cells (assayed by phalloidin staining). In comparison to DMSO control, Aβ_1–42_ exposure led to marked disruption of the mesh like actin assembly in cell clusters (Fig. 1f, panels i, iii). Higher magnification images showed in more detail that the fibril like actin mesh (DMSO) (Fig. 1f, ii) was absent in the Aβ_1–42_ cells (Fig. 1f, iv), in which the actin were mostly present in punctate clusters.

### ***ROR1 over expression abrogates Aβ***_***1–42***_*** induced degradation of cytoskeletal components***

**Figure 2:**
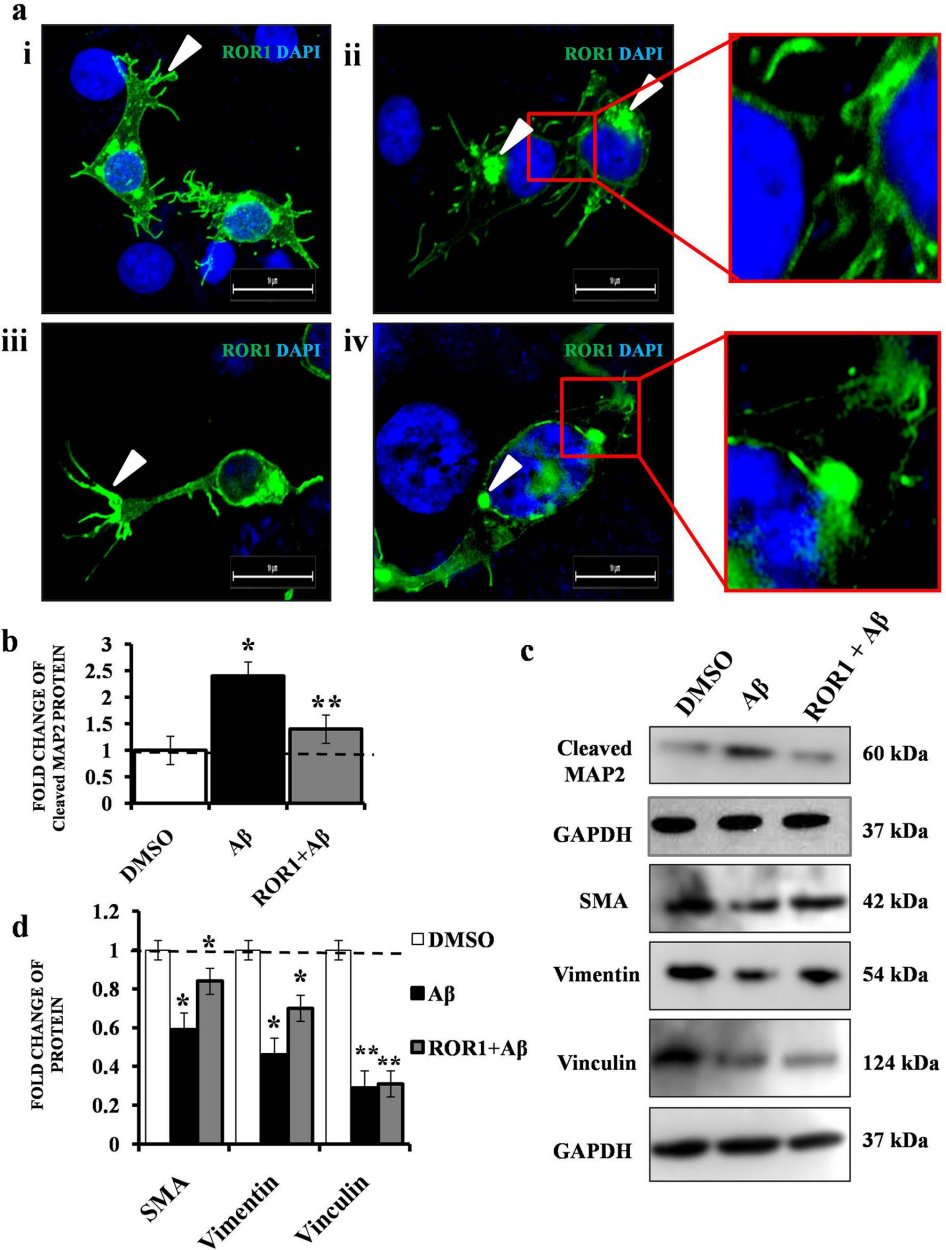
Consequences of ROR1 over expression on cytoskeletal components in Aβ_1–42_ treated cells. (**a**) Confocal microscopy images of SHSY-5Y cells transfected with ROR1-GFPSpark and stained with DAPI—panel i and iii. ROR1 over expression leads to aberrant terminal neurite outgrowths (white wedge); panel ii and iv. and insets. In dividing cells, ROR1 is localised to the cytokinetic bridge and terminal MTOCs (white wedge), Scale bars, 10 μm; For each confocal experiment, images of at least 30 cells (or cell fields) were captured and the experiments were repeated thrice (n = 3). (**b**) Graph depicting the mean value of optical density of Cleaved MAP2 bands, normalized against GAPDH. (**c**) Western blot (n = 3) showing the Cleaved MAP2, SMA, Vimentin, Vinculin and GAPDH levels in cells treated with DMSO (control), Aβ_1–42_ and ROR1 + Aβ_1–42_. (**d**) Graph depicting the mean value of optical density of SMA, Vimentin and Vinculin bands, normalized against GAPDH. Error bars indicate ± SD. Significance level between different experimental pairs is shown (NS, not significant; *p < 0.05; **p < 0.01; ***p < 0.001).

ROR1 having been down regulated in the study model, the next logical approach would be to see if ROR1 over expression produced significant phenotypic changes. Fluorescent confocal microscopy, 24 h post transfection with a ROR1-GFP Spark clone in SHSY-5Y cells, showed its sub-cellular distribution and marked alterations in the cellular structure (Fig. 2a., panels i, iii). A transient over expression of ROR1 led to the generation of multiple neurites in cells (marked with white arrows) limited to the cell terminals. Super-resolution microscopic images showed that in dividing cells, ROR1 was distinctly enriched in the cytokinetic bridge (Fig. 2a, panel ii and inset) and probably, the terminally located MTOCs (Fig. 2a, panel iv and inset, marked by white arrows). Following the observation that a transient increase in ROR1 promoted neurite generation, we could further show that ROR1 over expression prior to Aβ_1–42_ treatment hindered the cleavage of MAP2, indirectly indicating that ROR1 helped preserve the microtubule network (Fig. 2b,c). Similar changes were also observed in the SMA and Vimentin levels (Fig. 2c,d), but Vinculin did not show any significant recovery.

### ***Over expressed ROR1 promotes neurite elongation in presence of Aβ***_***1–42***_

**Figure 3:**
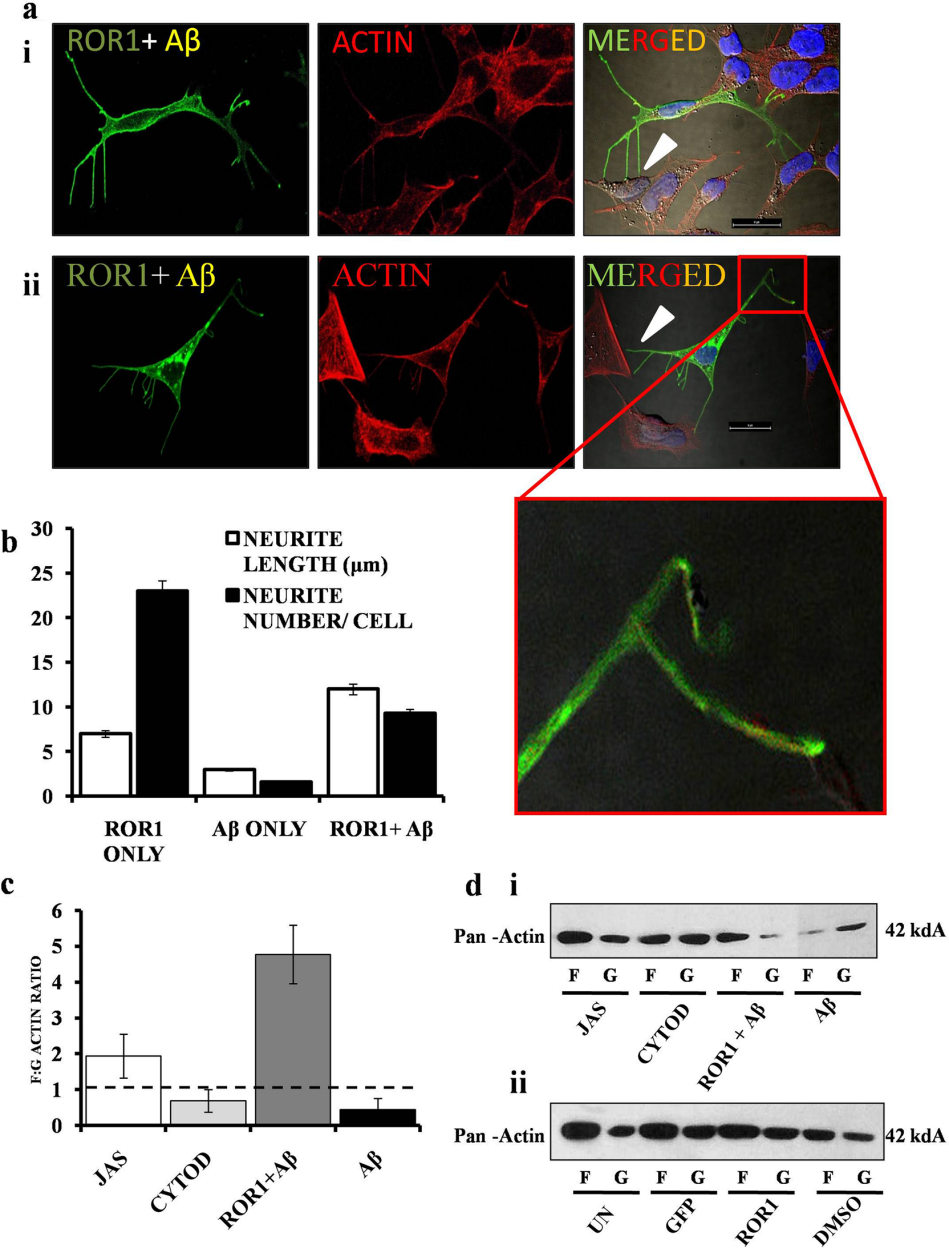
Effect of ROR1 on neurite elongation and actin ratio in Aβ_1–42_ treated cells. (**a**) Confocal microscopy images of SHSY-5Y cells transfected with ROR1-GFPSpark, treated with Aβ_1–42_ and stained with DAPI—panel i, ii and inset. ROR1 + Aβ_1–42_ leads to fewer but more elongated neurites which makes contact with adjacent cells (white wedge), Scale bars, 10 μm; For each confocal experiment, images of at least 30 cells (or cell fields) were captured and the experiments were repeated thrice (n = 3). (**b**) Graph depicting the mean value of neurite length and neurite numbers in ROR1, Aβ_1–42_ and ROR1 + Aβ_1–42_ cells. (**c**) Graph depicting the mean value of F: G actin ratio in Jasplakinolide, Cytochalasin-D, ROR1 + Aβ_1–42_ and Aβ_1–42_ treated cells, compared to their respective controls. (**d**) (i) Western blot (n = 3) showing the Pan -actin levels in the F and G fractions of cells treated with Jasplakinolide, Cytochalasin-D, ROR1 + Aβ_1–42_ and Aβ_1–42_. (ii) Western blot (n = 3) showing the Pan -actin levels in the F and G fractions of untreated cells, GFP transfected cells, ROR1 transfected cells and cells treated with DMSO. In each case, F: G ratio (y-axis) > 1. Error bars indicate ± SD. Significance level between different experimental pairs is shown (NS, not significant; *p < 0.05; **p < 0.01; ***p < 0.001).

We next investigated if the aberrant neurite generation due to ROR1 over expression could also occur on treatment of Aβ_1–42_, and if so, then how it would affect the cellular architecture. The following experiments were performed in the background of ROR1 over expression only, and additional controls like empty GFP were deemed counterintuitive. We found that a transient increase of ROR1 and Aβ_1–42_ led to an increase of neurites (Fig. 3a, panel i, ii and inset). However, unlike only ROR1 over expression, here, the neurites were significantly elongated in length, but less in number (Fig. 3b). In order to a get a better estimate of the neurite dynamics, we measured the ratio of neurite length: neurite number for the ROR1, Aβ_1–42_ and ROR1 + Aβ_1–42_ group, which showed that the ratio was highest ROR1 + Aβ_1–42_, followed by ROR1 and then only Aβ_1–42_ (Supplementary Fig. 2). Another interesting observation was that the elongated neurites were directed towards juxtaposed cells where they made contacts (marked by white arrows) and ROR1 was specifically enriched in the neurite terminals (Fig. 3a, panel ii inset). In order to better understand how ROR1 itself, or in conjunction with Aβ_1–42,_ were affecting the cytoskeletal dynamics, we performed a Filamentous: Globular (F: G) actin assay. On exposure to Jasplakinolide (actin stabiliser) the F: G ratio was > 1 (compared to DMSO control) (Fig. 3c, d(i)). Cytochalasin D (actin depolymeriser) had the reverse effect. Treatment with Aβ_1–42_ markedly decreased the ratio, but on prior increase with ROR1 followed by Aβ_1–42_, there was a strong enrichment of filamentous actin (or inhibition of actin depolymerisation) which led to a subsequent increase of the F: G Actin ratio (Fig. 3d (ii). for the control experiments and quantitation in Supplementary Fig. 3).

### ***Hsa-miR-146a-5p and 34a-5p are up regulated by Aβ***_***1–42***_*** and target ROR1 and Vimentin***

**Figure 4:**
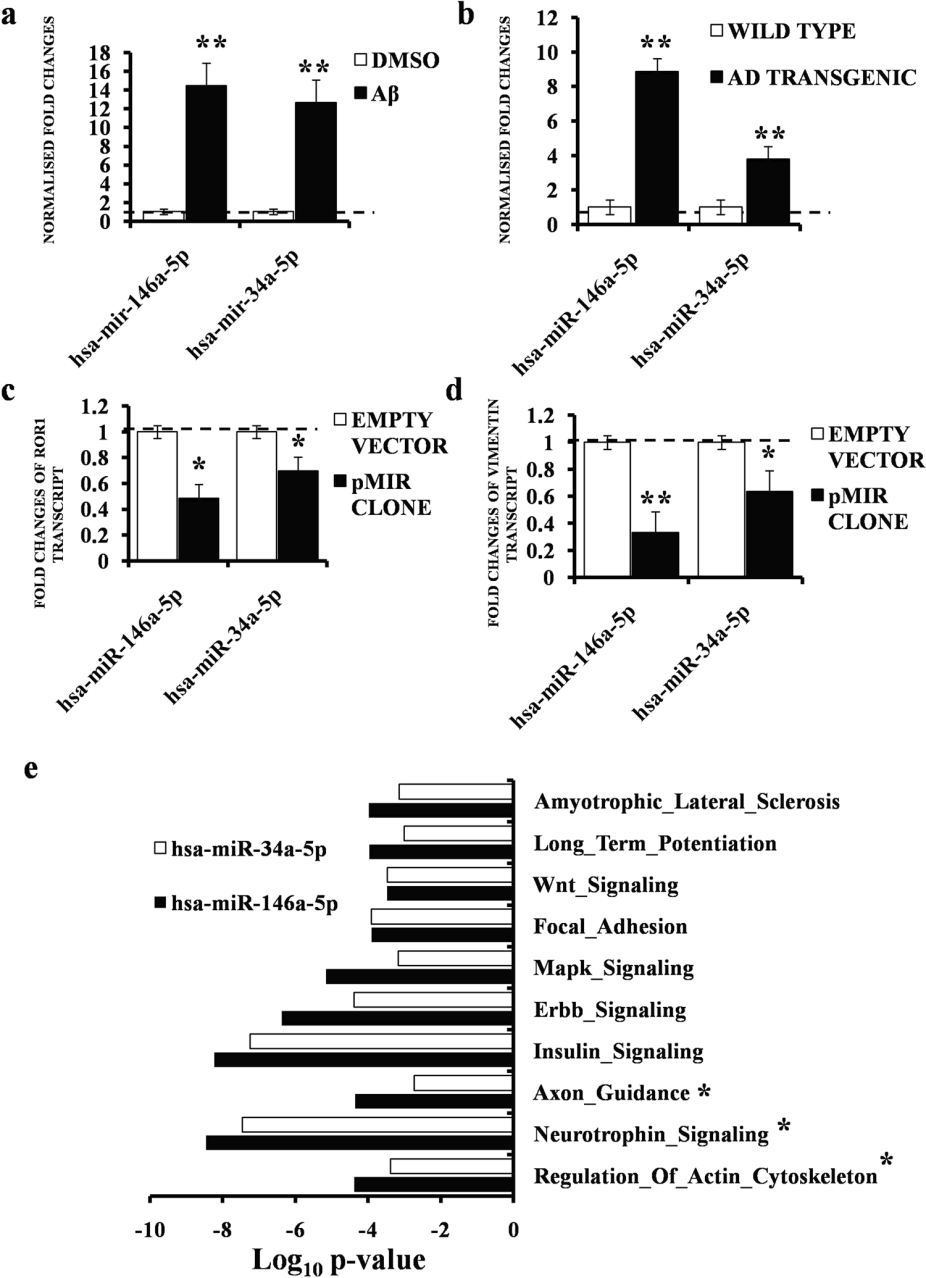
Dysregulation of hsa-mir-146a-5p, hsa-mir-34a-5p in Aβ_1–42_ treated cell model and mice AD model. (**a**) Graph depicting three (n = 3) independent biological replicates quantifying levels of hsa-mir-146a and hsa-mir-34a by qRT-PCR in SHSY-5Y cells treated with 1 µM Aβ_1–42_ or treated with only DMSO. (**b**) Graph depicting three (n = 3) independent biological replicates quantifying levels of hsa-mir-146a and hsa-mir-34a by qRT-PCR in transgenic AD mice or age matched wild type mice brain tissues. (**c**) Graph depicting three (n = 3) independent biological replicates quantifying levels of ROR1 by qRT-PCR in SHSY-5Y cells treated with hsa-mir-146a-5p and hsa-mir-34a-5p pMIR clones or corresponding empty vector controls. (**d**) Graph depicting three (n = 3) independent biological replicates quantifying levels of Vimentin by qRT-PCR in SHSY-5Y cells treated with hsa-mir-146a-5p and hsa-mir-34a-5p pMIR clones or corresponding empty vector controls. Levels of U6snRNA were taken as endogenous control for the miRNAs and levels of GAPDH were taken as endogenous control for the mRNA levels. The levels of individual miRNAs or mRNA were normalized by the corresponding U6snRNA or GAPDH levels. Fold changes were computed by considering the relative levels of lncRNA in corresponding controls to be 1. Error bars indicate ± SD. Significance level between different experimental pairs is shown (NS, not significant; *p < 0.05; **p < 0.01; ***p < 0.001). (**e**) KEGG analysis of the 2 de regulated miRNAs; bar graphs indicate Log (*p* value) and ranges from (-)10 to 0. Pathways deregulated in AD are marked with an asterisk.

To gain a mechanistic insight into the trigger of ROR1 deregulation in Aβ_1–42_ treated cells, we pursued the regulatory RNA-protein network model and looked for miRNA interacting (and preferably repressing) components of ROR1 network using ENCORI (http://starbase.sysu.edu.cn/index.php). The miRNA-mRNA tool was used with the following parameters—predicting program—5, miRNA-mRNA with Pan-Cancer analysis-2 and stringency of CLIP data-3. With these attributes, ROR1 was predicted to interact with hsa-miR-146a-5p and hsa-miR-34a-5p (Supplementary Fig. 4). The same bioinformatics search predicted that that these two miRNAs also targeted a cytoskeletal protein of our interest—Vimentin. qRT-PCR, with primers designed against the human mature miRNA sequences showed that hsa-miR-146a was more abundant compared to hsa-miR-34a, (normalised against control U6snRNA) (Supplementary Fig. 5). Both the miRNAs were strongly and significantly up-regulated in the Aβ_1–42_ treated cell model (Fig. 4a). Subsequent assays using AD transgenic mice brain tissues revealed almost the same patterns of up regulation, although here, the fold change of increase of hsa-miR-146a was much greater than hsa-miR-34a (Fig. 4b). In order to validate the bioinformatics prediction, we transiently over expressed both the miRNAs individually (using miRNA clones in pMIR vector) and then looked at the transcript levels of ROR1, and indeed both of them targeted and strongly repressed ROR1 levels (Fig. 4c), although the effect of hsa-miR-146a-5p was more pronounced. These two ROR1 targeting miRNAs also targeted and repressed Vimentin (Fig. 4d), validating the prediction data. Combining both the results, we found that hsa-miR-146a-5p was the stronger common repressor of both these proteins. Looking at the effect of these two miRNAs on cytoskeletal proteins, we posited that they would be involved in neurological processes which are governed by such components. Hence, we performed a Gene Enrichment analysis with the help of DIANA tools (miRPath module). Intuitively, GSEA revealed that both hsa-miR-146a and hsa-miR-34a were involved in core neurological pathways like Long Term Potentiation, Wnt signalling, Insulin signalling and Mapk signalling pathways (Fig. 4e). However, they were more enriched in the processes like—Regulation of actin cytoskeleton, Neurotrophin signalling and axon guidance, all of which were deregulated in AD. In this analysis too, hsa-miR-146a showed a stronger enrichment compared to hsa-miR-34a (Fig. 4e).

### LncRNA NEAT1 exerts a protective effect by repressing miR146a and miR-34a

**Figure 5:**
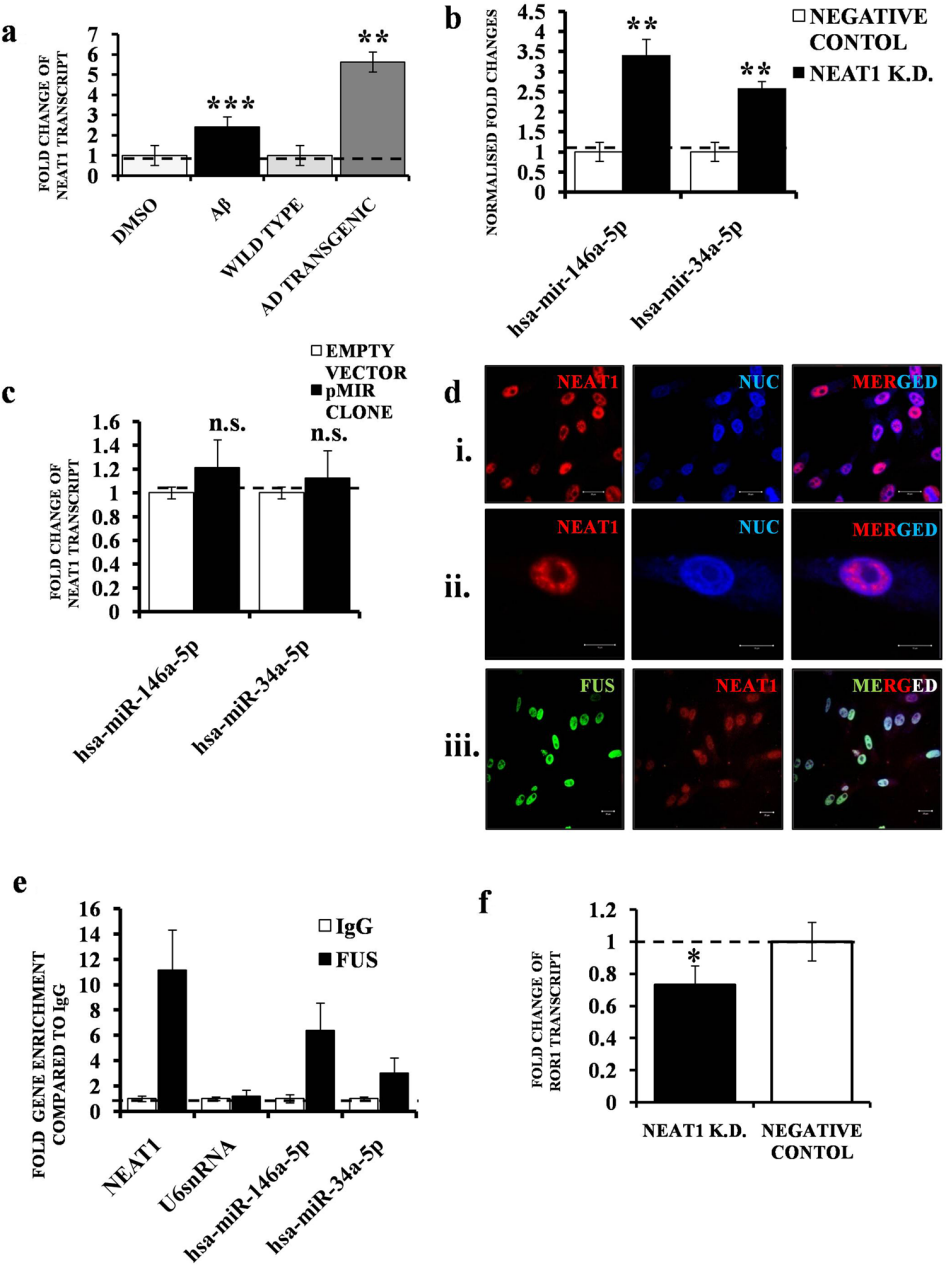
Endogenous interaction and regulation of miR146a and miR-34a by NEAT1 in SHSY5Y cells. (**a**) Graph depicting three (n = 3) independent biological replicates quantifying levels of NEAT1 by qRT-PCR in SHSY-5Y cells treated with 1 µM Aβ_1–42_ or treated with only DMSO; wild type and AD transgenic mice. (**b**) Graph depicting three (n = 3) independent biological replicates quantifying levels of miR146a and miR-34a by qRT-PCR in SHSY-5Y cells treated with NEAT1 siRNA or corresponding negative control. (**c**) Graph depicting three (n = 3) independent biological replicates quantifying levels of NEAT1 by qRT-PCR in SHSY-5Y cells treated with hsa-mir-146a-5p and hsa-mir-34a-5p pMIR clones or corresponding empty vector controls. (**d**) RNA-FISH assay of NEAT1 in SHSY5Y cells showing its enrichment in the areas of the nucleus distinct from the nuclear stain DRAQ5®; panel (i) Low magnification (1x) image of a cell population. Scale bars, 20 μm; panel (ii). High magnification (4x) image of a single nucleus distinct NEAT1 paraspeckles. Scale bars, 5 μm; panel (iii). Co localization Analysis—Sequential Immunocytochemistry (ICC) and RNA Fluorescence In Situ Hybridisation (FISH) assay of NEAT1 combined with FUS in SHSY-5Y cells—Low magnification (1x) image of a cell population. Scale bars, 20 μm. For each FISH or combined ICC-FISH experiment, images of at least 30 cells (or cell fields) were captured and the experiments were repeated thrice (n = 3). (**e**) Graph depicting three (n = 3) independent biological replicates quantifying enrichment of NEAT1, U6snRNA, hsa-mir-146a-5p and hsa-mir-34a-5p by qRT-PCR in SHSY-5Y cells after pull down with FUS antibody or control IgG antibody in RIP Assays. Levels of U6snRNA were taken as endogenous control. The levels of individual miRNAs or lncRNA were normalized by the corresponding U6snRNA levels. Fold change was calculated by considering the relative levels of miRNAs or lncRNA in scrambled controls to be 1. (**f**) Graph depicting three (n = 3) independent biological replicates quantifying levels of ROR1 by qRT-PCR in SHSY-5Y cells treated with NEAT1 siRNA or corresponding negative control. Error bars indicate ± SD. Significance level between different experimental pairs is shown (NS, not significant; *p < 0.05; **p < 0.01; ***p < 0.001).

**Figure 6:**
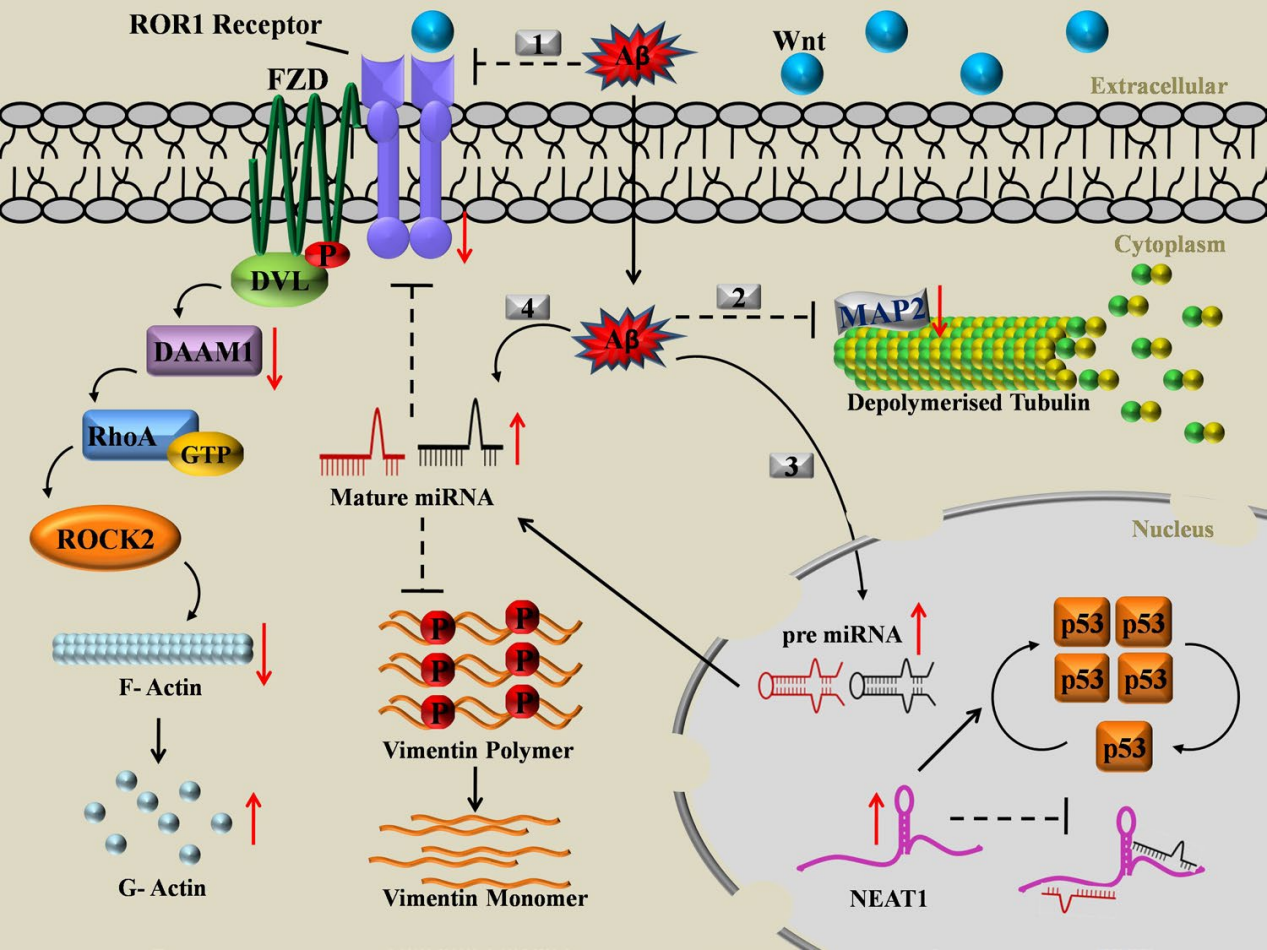
Graphical Abstract: Cartoon representation of the molecular events cascade on exposure to Aβ_1–42_ in neuronal cells. Aβ_1–42_ has multifaceted effects on cells-1. Extracellular Aβ_1–42_ inhibits the levels of microtubule associated RTK ROR1, both at the transcript and protein levels thereby affecting the planar cell polarity pathway leading to the alteration of F:G actin dynamics. 2. Cytosolic Aβ_1–42_ affects microtubule dynamics by deregulation of MAP2. 3. Nuclear translocated Aβ_1–42_ up regulates the precursor miRNAs and NEAT1. Up regulated NEAT1 in the nucleus exerts a protective role by repressing miRNAs and stabilising p53. 4. The compounded effect of cytosolic and nuclear Aβ_1–42_ affects the mature miRNA pool, which targets and co-represses ROR1 and Vimentin.

Continuing with the ncRNA regulatory networks governing ROR1, we introduced another layer of complexity. We used the ENCORI database to look for the potential lncRNA interactors of hsa-miR-146a-5p and hsa-miR-34a-5p. We employed the miRNA-lncRNA tool which had data from Ago-CLIP seq experiments and predictive data from miRanda, with the search parameters-CLIP data, high stringency (≥ 3) and Degradome data, high stringency (≥ 3). Following these search criteria, hsa-miR-146a-5p and hsa-miR-34a-5p were predicted to interact with a lncRNA –NEAT1 (Supplementary Fig. 6). In order to implicate NEAT1 in AD, we first looked for its deregulation in our cell model and mice model, with human and mouse primers, respectively. NEAT1 transcript levels were up regulated in both disease models (Fig. 5a), but the mice model showed a stronger increase, probably hinting at the fact that the transgenic mice AD model better mimicked late stage AD. Having ascertained that NEAT1 indeed was deregulated in AD, to validate the prediction data, we transiently knocked down NEAT1 levels (using siRNA) and checked for the down regulation (Supplementary Fig. 7). We probed for the levels of hsa-miR-146a and hsa-miR-34a after NEAT1 knock down. Compared to a negative control siRNA, treatment with NEAT1 siRNA led to a concomitant increase of both the miRNAs, with hsa-miR-146a showing a higher increase (Fig. 5b). Conversely, we also tested if this putative interaction and suppression was bi-directional. A transient over-expression of the mature miRNA clones in cells (Fig. 5c) failed to elicit a response in the NEAT1 levels. We employed combined Immunocytochemistry (ICC) plus RNA –Fluorescence In Situ Hybridisation (RNA FISH), and RNA Immuno Precipitation (RIP) to study lncRNA-miRNA interaction. NEAT1 lncRNA was observed in nuclear locations different from that of the DNA marker, in cell populations (Fig. 5d, panel i). A higher magnification image (Fig. 5d, panel ii) showed its distinct distribution in defined spots called nuclear paraspeckles. Further, NEAT1 was predicted to interact with a RNA Binding protein (RBP) FUS using the lncRNA-RBP tool from ENCORI (Supplementary Fig. 8). Combined ICC of FUS with RNA FISH (Fig. 5d, panel iii), using NEAT1 specific probes, showed a strong overlap between the two in the cell nucleus. From the theoretical prediction and co-localisation analysis, we next designed a RIP experiment using FUS as the bait. Compared to control IgG, FUS pull down from cell lysates and subsequent assay by qRT-PCR showed a strong enrichment of NEAT1 (Fig. 5e). A reanalysis using mature miRNA specific probes from the same FUS pull down RNA also subsequently showed a clear enrichment of the NEAT1 interacting miRNAs—hsa-miR-146a and hsa-miR-34a. In the RIP assay, hsa-miR-146a showed near double enrichment compared to hsa-miR-34a, indicating that the former had a stronger interaction with NEAT1. In order to validate that the effect of NEAT1 knock-down was not just restricted to the miRNA levels, but their target ROR1 as well, we looked at the transcript levels of ROR1 after NEAT1 silencing (Fig. 5f) and indeed, ROR1 levels went down significantly on transient NEAT1 suppression, thereby confirming the hypothesis that NEAT1, its interacting miRNAs and their target ROR1, essentially constitute a single entity (Fig. 6).

## Discussion

In this study, we focus on ROR1 with the motivation that cytoskeleton disruption in AD due to Aβ_1–42_ is a well recognised hallmark^5–9^ and we could establish the same through biochemical assays and confocal imaging. Further, in recent times, microtubule associated ROR1 has been implicated in reinforcement of neuronal network^15–18^, which we find to be true on ROR1 over expression and subsequent neuritogenesis with the caveat that AD involves significant disruption of the same. ROR1 was specifically localised in the cytokinetic bridge and MTOCs. However, use of specific microtubule markers would confirm this hypothesis. But, as we wanted to look at the effects of ROR1 in AD (which affects post-mitotic neurons), this line of investigation was not pursued. Intuitively, we find ROR1 levels to decrease in our AD model. The same transient over expression of ROR1 in presence of Aβ_1–42_ is found to be necessary and sufficient to hamper cytoskeletal degradation of key proteins, promote neuritogenesis and drastically alter the F: G actin dynamics. In search for the small molecule regulators of ROR1, we could identify two miRNAs—miR-146a and miR-34a, which were theoretically predicted to target ROR1. Subsequent validation in our cell model and transgenic mice AD model revealed significant up regulation of both. Using mature miRNA clones, we substantiated the hypothesis that both miR-146a and miR-34a targeted and repressed ROR1 levels in cells, miR-146a being the stronger repressor. Fortuitously, both of these also targeted Vimentin, a cytoskeletal protein of importance in AD. It was surprising that the repression of Vimentin was in fact stronger than ROR1, which leads us to believe that up regulation of these two miRNAs cumulatively affects the cytoskeleton disruption in AD by dual repression of ROR1 and Vimentin. It is however important to note that the decrease of ROR1 levels might also act due to a transcriptional repression pathway and not just by repression by miRNAs, which would be part of a future study. It is not surprising, therefore, to find that miR-146a and miR-34a are parts of core neurobiological pathways implicated in AD, like LTP, axon guidance and regulation of actin cytoskeleton. These novel results are also backed up by literature reports that show miR-146a and miR-34a govern the regulators of actin pathways, namely RhoA and ROCK1^22–24^. To further understand how these miRNAs were themselves regulated, we deciphered their interaction with the lncRNA NEAT1, which recently has been shown to be deregulated in a plethora of neurodegenerative scenarios^46–49^. Using the same AD cell and mice model, we validated the up regulation of NEAT1. The direct interaction of miR-146a and miR-34a with NEAT1 was characterised with subsequent transient knock down, RIP and combined ICC with RNA-FISH experiments. We could also show a direct repercussion of perturbation of the NEAT1 level on ROR1 transcript levels, completing the proposed RTK-miRNA-lncRNA regulatory loop. Such repressive effects of lncRNAs on miRNAs have been shown recently in colorectal cancers^52^, pancreatic cancer^53^ and cardiomycetes apoptosis^54^.

Moreover, it was also found that the protein component of the miRNA machinery, Ago2, co-localised strongly with the NEAT1 interactor FUS in distinct nuclear clusters (Supplementary Fig. 9, Panels i and ii) pointing to the fact that the miRNA-lncRNA machinery possibly interact in a closed loop. Such instances of the nuclear shuttling of Ago2 have also been reported^55,56^.

Although there is evidence of the disruption of the cytoskeletal machinery in Alzheimer’s Disease due to the Aβ_1–42_ (and its effects on the proteins α-Tubulin, Vimentin and SMA)^57,58^, to our cognizance, this is the first consolidated network study to undisputedly connect ROR1 to Aβ_1–42_ treatment in AD, by showing its deregulation both at the transcript and protein levels. In-vivo over expression of ROR1 exerts protective effects on the gross cytoskeletal assembly and neurite formation. A functional link between ROR1 and its targeting miRNAs is established. Eventually, we could also show a regulatory paradigm of ROR1-miRNA 146a/34a—NEAT1 in AD.

## Conclusion

To summarise, effect of Aβ_1–42_ on cells were diverse (Fig. 6). First, Aβ_1–42_ deregulated the expression of RTK ROR1 and relevant cytoskeleton associated components. Second, cytosolic Aβ_1–42_ affected the mature miRNAs-miR-146a and miR-34a (through a hitherto unknown mechanism), which in turn repressed ROR1 and finally, nuclear Aβ_1–42_ differentially regulated NEAT1, which in turn regulated the miRNAs. Comprehending this RTK-miRNA-lncRNA network promises to initiate further studies involving other RTKs as potential therapeutic targets in abrogating AD pathophysiology.

## Materials and methods

### Ethics statement

All animal experiments were conducted following the institutional guidelines for the use and care of animals and approved by the Institutional Animal and Ethics Committee of the National Brain Research Centre (NBRC/IAEC/2012/71) and carried out in compliance with the ARRIVE guidelines.

### Cell culture and transfection

Human neuroblastoma cell lines SHSY-5Y were procured from NCCS, Pune, India and were cultured routinely in DMEM-F12 (Gibco) supplemented with 10% (v/v) heat-inactivated FBS (Gibco), antibiotics penicillin/streptomycin PS 1% (v/v) and 400 μg/ml G418 (Invitrogen, USA) at 33 °C in humidified condition and 5% CO_2_. All transfections were carried on 70–80% confluent cells using Lipofectamine 2000 (Invitrogen) as per manufacturer’s protocol. Unless otherwise mentioned, for single transfection experiment 1 µg (30 mm plate), 2.5 μg (60 mm plate) or 5 μg (100 mm plate) of plasmid DNA constructs as well as 5 μl, 10 μl or 15 µl of Lipofectamine 2000 respectively were used. Normalisation of transfection was performed using pEGFP-C1 (Clontech) and using the same protocol above followed by quantification of GFP positive cells using a microscope.

### Constructs, reagents and siRNAs

Constructs: Human ROR1 ORF mammalian expression plasmid, C-GFPSpark tag (HG13968-ACG); Reagents: Cytochalasin D—(ab143484), Jasplakinolide—(ab141409), DRAQ5™ (ab108410); siRNA: 4 siRNAs for Entrez gene 283131 for NEAT1: SI05189765 (FlexiTube siRNA), SI05189758, SI05189751, SI03682126, Product no: 1027416, Cat no: GS283131, Negative control siRNA (1022076).

### Aβ_1–42_ treated cell model

Lyophilised Aβ_1–42_ protein fragment (Sigma, A980) was weighed and reconstituted in DMSO to make a stock of 2 µM. From this stock, the requisite amount of Aβ_1–42_ was added to the petri-dishes (0.5 µl for 35 mm, 1 µl for 60 mm and 2 µl for 90 mm plates) to make the working concentration of 1 µM and the cells were exposed to this Aβ_1–42_ for 24 h. Only DMSO in the same concentration and amount was used as a control.

### APP/PS1 mice

Trangenic AD mice (APP/PS1 or B6C3-Tg (/APPswe, PSEN1dE9/) 85Dbo/J) were procured from the Jackson Lab. AD transgenic mice have human APPswe mutations (at positions K670N and M671L) and human presenilin gene with exon 9 deletion 1(PSEN1dE9) under a mouse prion gene promoter. Mice were supplied with water and food as often as necessary. AD mice, along with controls at their age of 12 months, were anaesthetized with xylazine (10 mg/kg body weight) and ketamine (100 mg/kg body weight) and perfused transcardially with PBS followed by 4% paraformaldehyde (w/v) in PBS. Brains were collected and further placed in 4% paraformaldehyde for 24 h and then treated with 10, 20 and 30% sucrose (in PBS) followed by sectioning in a cryo-microtome (20 μm thickness).

### Isolation of RNA from cells and FFPE tissue

Total RNA was extracted following manufacturer’s protocol using TriZol Reagent (Invitrogen, USA). We isolated RNA from paraffin-embedded tissue samples of AD mice; along with controls. In brief, isolation method for RNA from paraffin-embedded tissues consists of the following steps: De-paraffinization: For RNA extraction from tissue sections obtained from AD mice or controls, two sections (biological replicates, 20 µm thick) were put in 1.5 ml tubes, deparaffinized by double xylene washes (5 min each) followed by two centrifugations at room temperature (10,000 g, 10 min). Rehydration: The supernatant was discarded and the pellets were washed with absolute ethanol (1 ml) and 95% ethanol (1 ml) in DEPC water (successively). Following each step, the tissue was pelleted (10,000 g, 10 min). For digesting tissue proteins, following the final wash, alcohol was decanted; the pellets were dried in a drybath at 37 °C and put in 500 µl of digestion buffer (10 mMNaCl, 500 mMTris, pH 7.6, 20 mM EDTA and 1% SDS). Tissue proteins were removed using proteinase K (500 µg/ml) followed by incubation at 45 °C (16–18 h). Before RNA isolation, proteinase K was deactivated at 100 °C (7 min). RNA extraction: Total RNA was then isolated from tissues employing Trizol reagent as per manufacturer's instructions. Total RNA concentration was quantified by Nanodrop spectrophotometer (Thermofisher Scientific, USA). Details about tissues and their nanodrop concentrations are given in the Supplementary Table 1.

### Quantitative real-time PCR

2 μg total RNA was treated by DNase (Sigma) followed by cDNA preparation by primers (oligo dT or random hexamers), dNTPs and Reverse transcriptase (Fermentas). qRT-PCR was performed using Sybr green 2X Universal PCR Master Mix (ABI) in StepOne Real-time PCR system (ABI). For each gene, NTC was used at the same condition to determine the baseline and threshold value. Corresponding C_t_ values were used for the relative quantification (fold change) of a target gene in a sample compared to the parental cell is expressed in terms of 2^−ΔΔCt^ values after normalizing w.r.t. house-keeping gene (internal control).

### Gene-specific primers

PCR primers designed for and employed in this study are listed in Supplementary Table 2.

### RNA immunoprecipitation (RIP) assay

RNA Immunoprecipitation was performed on fixed cells (4% formaldehyde) following the Abcam RIP protocol (https://www.abcam.com/protocols/RIP) following the manufacturer’s instruction with modifications. SHSY5Y cells were harvested by trypsinization and resuspended in PBS, freshly prepared nuclear isolation buffer (1.28 M sucrose, 40 mM Tris–HCl, pH 7.5, 20 mM MgCl_2_, 4% Triton X-100) and water, and kept on ice for 20 min with frequent mixing. Next, a centrifugation step (2,500 G, 15 min) was performed to pellet the nuclei. Then the nuclear pellet was resuspended in freshly prepared RIP buffer (150 mM KCl, 25 mM Tris pH 7.4, 5 mM EDTA, 0.5 mM DTT, 0.5% NP40, 100 U/ml RNAase inhibitor, Protease inhibitors). The nuclei fraction was sonicated with the following parameters—30% amplitude—10 s—1 min gap, and the process was repeated 4 times. Following this, the solution was nutated at 20 rpm for 90 min at 4 °C. After nutation, the lysate was centrifuged at 12,000 RCF for 20 min at 4 °C. The pellet was discarded and the supernatant was used for protein estimation using Bradford reagent. 5 mg of total protein in RIP buffer was used for each sample to which 3 μg of antibody (FUS or IgG) was added and incubated at 4 °C with gentle rotation overnight. After that, to each tube 40 μl protein A/G beads were added and incubated for 2 h at 4 °C with gentle rotation. Then, beads were pelleted by centrifugation at 2,500 rpm for 30 s, the supernatant was removed, and the beads resuspended in 500 μl RIP buffer. Beads were washed for a total of three RIP washes, followed by one wash in PBS. The co precipitated RNAs were isolated by resuspending beads in TRIzol RNA extraction reagent (1 ml) according to manufacturer’s instructions. RNA was collected in 15 μl DEPC water. The total RNA was used to make cDNA with Random Hexamer or miRNA specific stem loop primers. The following steps were same as the Quantitative Real-time PCR (qRT-PCR) protocol stated above.

### Sequential immunocytochemistry (ICC) and RNA fluorescence in situ hybridisation (FISH) assay

Sequential Immunocytochemistry (ICC) and RNA Fluorescence In Situ Hybridisation (FISH) Assay was performed following Stellaris® RNA FISH protocol according to the manufacturer’s instruction, with modifications. Briefly, SHSY5Y cells were seeded on 18 mm coverglass in a 35 mm cell culture plate. The growth medium was aspirated, and washed with 1 ml of 1X PBS. 1 ml of fixation buffer (3.7% (vol/vol) formaldehyde in 1X PBS) was added and incubated at room temperature for 10 min. Then cells were washed twice with 1 ml of 1X PBS. To permeabilize, cells were immersed in 1 ml of 0.1% Triton X-100 in 1X PBS for 5 min at room temperature followed by washing with 1 ml of 1X PBS. Then, the cells on cover glass were inverted on 100 μl of appropriately diluted (1:100) primary antibody (anti-FUS antibody) in 1X PBS and incubated at 4 °C overnight. Following this, cells were inverted again in culture plate and washed with 1 ml of 1X PBS for 10 min, and repeated 2 more times. Next, 1 ml secondary antibody (1:300) in 1X PBS was added and kept at room temperature (2 h, in the dark). Again they were washed with 1 ml of 1X PBS for 10 min, and repeated 2 more times. Then, 1 ml of fixation buffer was added and incubated at room temperature for 10 min followed by two washes 1 ml of 1X PBS. The 1X PBS was aspirated off the cover glass containing adherent cells within the 35 mm plate. 1 ml of Wash Buffer A was added, and incubated at room temperature for 2–5 min. Within a humidified chamber, 100 μL of the Hybridization Buffer containing probe (Human NEAT1 with Quasar® 570 dye) onto the Parafilm was dispensed and the cover glass was gently transferred, cells side down, onto the 100 μL drop of Hybridization Buffer containing probe. The humidified chamber was covered with the tissue culture lid, and sealed with Parafilm. Cells were incubated in the dark at 37 °C for 16 h. Then the cover glass was gently transferred, cells side up, to a fresh 35 mm plate containing 1 ml of Wash Buffer A and incubated in the dark at 37 °C for 30 min. The Wash Buffer A was aspirated, and then 1 ml of DAPI nuclear stain (Wash Buffer A consisting of 5 ng/ml DAPI) was added to counter stain the nuclei followed by incubation in the dark at 37 °C for 30 min. The DAPI staining buffer was aspirated, and then 1 ml of Wash Buffer B was added with incubation at room temperature for 2–5 min. Finally, a small drop (approximately 15 μl) of Vectashield Mounting Medium was added onto a microscope slide, and the cover glass was mounted onto the slide, cells side down. Excess anti-fade from the perimeter of the cover glass was gently wicked away. The cover glass perimeter was sealed with clear nail polish, and allowed to dry.

### Western blot

Phosphate buffer saline (PBS) washed pellet from cell lines were lysed on on ice in lysis buffer (1 M Tris–HCl, pH 7.5, 1 N NaCl, 0.5 M EDTA, 1 M NaF, 1 M Na_3_VO_4_, 10% SDS, 20 mM PMSF, 10% Triton X-100, 50% glycerol) for 30 min in presence of complete protease inhibitor (Roche Diagnostics) and centrifuged at 13,000*g* for 15 min. Protein amounts were quantified using Bradford spectrophotometric assay.

The cell lysate was separated on SDS gel according to molecular weight then it was transferred to PVDF membrane (Millipore Corporation) which was blocked by 5% skimmed milk in TBST (50 mMTris-HCl, 150 mM NaCl, pH 7.5 containing 0.05% Tween 20). After that membrane was probed with primary antibody, followed by the incubation with HRP conjugated secondary antibody. The membranes were then developed with ECL kit (Pierce or Abcam). Band intensities were measured by Quantity One (Bio-Rad). Experiments were repeated thrice. Significance testing (*p* values) was performed by unpaired *t*-test.

### Antibodies

Mouse monoclonal anti-ROR1 (ab91187)—1:500; Rabbit polyclonal anti-α-tubulin (ab24246)—1:3000; Mouse monoclonal anti-SMA (ab7817)—1:2000; Rabbit monoclonal anti-Vimentin (ab92547)—1:2000; Mouse monoclonal anti-MAP2 (ab11267)—1:1000; Rabbit monoclonal anti-Vinculin (ab219649)—1:2000; Rabbit monoclonal anti-GAPDH (ab181602)—1:3000; Mouse monoclonal Anti-Actin (Pan) antibody [C4] (ab14128)—1:2000; Rabbit monoclonal anti-TLS/FUS (ab243880)—1:100 (for ICC), 3 μg total (for RNA Immunoprecipitation); Mouse monoclonal anti-Argonaute-2 (ab57113)—1:100 (for ICC); Goat anti-mouse IgG (Alexa Fluor 488) (ab150113)—1:200 (for ICC); Goat anti-mouse IgG (Alexa Fluor 568) (ab175473)—1:200 (for ICC); Goat anti-mouse IgG (Alexa Fluor 647) (ab150115)—1:200 (for ICC); Goat anti-rabbit IgG (Alexa Fluor 488) (ab150077)—1:200 (for ICC); Goat anti-rabbit IgG (Alexa Fluor 568) (ab175471)—1:200 (for ICC); Goat anti-rabbit IgG (Alexa Fluor 488) (ab150079)—1:200 (for ICC).

### Immunocytochemistry

Immunocytochemistry was performed on fixed cells following the abcam ICC protocol (https://www.abcam.com/protocols/immunocytochemistry-immunofluorescence-protocol) following the manufacturer’s instruction with slight modifications. Briefly, cells were fixed using 4% paraformaldehyde in PBS pH 7.4 for 12 min at room temperature. This was followed by cell washes in chilled PBS (thrice). Cells were then permeabilized with 0.1–0.25% Triton X-100 for 10 min at room temperature followed by PBS washes for three times for 5 min. Cells were then blocked with 1% BSA, 22.52 mg/ml glycine in PBST (PBS + 0.1% Tween 20) for 30 min to block unspecific binding of the antibodies. Then, the cells were incubated with diluted primary antibody in 1% BSA in PBST in a humidified chamber for overnight at 4 °C. The solution was decanted and the cells washed three times in PBS, 5 min each wash. Cells were then incubated with the secondary antibody in 1% BSA for 1 h at room temperature in the dark, following which the secondary solution was drained and cells washed in PBS (thrice, 5 min each in the dark). Finally, the cells were incubated with 0.1–1 μg/ml DAPI (DNA stain) for 5 min. The DAPI solution was discarded and cells were rinsed twice with PBS. Coverslips were mounted on fresh, cleaned and dried slides with a drop of mounting medium and sealed with nail polish to prevent drying and movement under microscope.

### F/G actin assay

After appropriate treatments, cells were scrapped from the petri dishes and washed twice in PBS. Cells were then centrifuged at 800 RCF for 3 min at 4 °C. The cell pellets were then resuspended in 200 μl PBS with 0.1% Triton-X-100 (with protease inhibitors). After incubation for 15 min with slight agitation, cells were again centrifuged at 15,000 RCF at 4 °C for 5 min. The soluble supernatant (which contained G-actin) was separated and the Triton-X-100 insoluble pellet (predominantly F-actin) was resuspended in 200 μl RIPA buffer. The soluble and insoluble fractions were mixed with 5X Loading dye, heated at 98 °C for 10 min, equal volumes of the two fractions were loaded and separated on a 12% SDS gel using standard electrophoresis protocol. Actin levels were assayed using the pan actin antibody (Clone C4).

### Trypan blue exclusion assay

For the Trypan Blue Exclusion Assay, SHSY-5Ycells were seeded in 6 well clear bottom plates (Thermo). After the appropriate treatments, media was discarded from cell cultures and washed twice with PBS. Cells were trypsinised and resuspended in 1 ml fresh media (without FBS). Following this, the experiment was performed as per manufacturer’s instruction. Cell viability was plotted as % viability compared to controls.

### MTT assay

For the MTT cell viability assay, standard protocols were followed as per the manufacturer’s instruction. Cell viability was plotted as % viability compared to controls.

### Gene set enrichment analysis and interaction predictions

GSEA, miRNA target analysis and miRNA-lncRNA predictions were performed as outlined in^57^.

### Statistical analysis

Statistical analysis and significance testing were performed as given in^57^.

### Consent for publication

The authors declare that they give consent for publication with the contents of this article.

## Supplementary Information

Supplementary Information.

## Data Availability

The authors declare that they consent to make the data and materials available upon request.

## Abbreviations

Aβ: Amyloid β peptide
RTK: Receptor Tyrosine Kinase
miRNA: Micro RNA
lncRNA: Long non coding RNA
RBP: RNA Binding Protein
