# Supplementary material for: Receptor tyrosine kinase ROR1 ameliorates Aβ1–42 induced cytoskeletal instability and is regulated by the miR146a-NEAT1 nexus in Alzheimer’s disease
Source: Sci Rep. 2021 Sep 28;11:19254. doi: 10.1038/s41598-021-98882-0 (PMC8479066; doi:10.1038/s41598-021-98882-0)
Supplement: Supplementary file 1 — Supplementary Information. [file 41598_2021_98882_MOESM1_ESM.docx]

**Receptor Tyrosine Kinase ROR1 ameliorates Aβ_1-42_ induced cytoskeletal instability and is regulated by the miR146a-NEAT1 nexus in Alzheimer’s Disease**

Kaushik Chanda^1^, Nihar Ranjan Jana ^2, 3^ and Debashis Mukhopadhyay^1^, ^†^

**SUPPLEMENTARY DATA**

**Supplementary Table 1: Quantification of RNA samples using Nanodrop.**

| **SR NO.** | **SAMPLE ID** | **NANODROP READINGS (ng/μl)** | **NANODROP READINGS (260/280)** | **NANODROP READINGS (260/230)** | **REMARKS** |
| --- | --- | --- | --- | --- | --- |
| 1 | WILD TYPE 1 | 858.4 | \| 1.8 \| 1.99 \| \| --- \| --- \| | 1.99 | QC PASS |
| 2 | WILD TYPE 2 | 769.35 | 1.88 | 1.98 | QC PASS |
| 3 | WILD TYPE 3 | 824 | 1.9 | 2.01 | QC PASS |
| 4 | AD TRANSGENIC 1 | 687.3 | 1.85 | 2.03 | QC PASS |
| 5 | AD TRANSGENIC 2 | 242 | 1.82 | 1.94 | QC PASS |
| 6 | AD TRANSGENIC 3 | 469.6 | 2.1 | 1.89 | QC PASS |

**Supplementary Table 2: PCR primers used to validate the expression of mature miRNAs, mRNA, lncRNA and control gene.**

| **Primer name** | **Sequence (5’-3’)** |
| --- | --- |
| 146a-5p_STEM_LOOP | CTC AAC TGG TGT CGT GGA GTC GGC AAT TCA GTT GAG AAC CCA TG |
| 146a-5p _RT_FWD | ACA CTC CAG CTG GGT GAG AAC TGA ATT CCA |
| 34a-5p_STEM_LOOP | CTC AAC TGG TGT CGT GGA GTC GGC AAT TCA GTT GAG ACA ACC AG |
| 34a-5p _RT_FWD | ACA CTC CAG CTG GGT GGC AGT GTC TTA GCT |
| Universal Reverse Primer | CCA GTG CAG GGT CCG AGG TA |
| Mouse_NEAT1_FORWARD | GTGAAGACACAACAGCCTTTCTCC |
| Mouse_NEAT1_ REVERSE | GCTCTGGGACCTTCGTGACTCT |
| Mouse_U6 snRNA_FORWARD | TGCAGTGTGCCAATGTTTCG |
| Mouse_U6 snRNA_REVERSE | CGCTTCGGCAGCACATATAC |
| Human _NEAT1_FORWARD | GTATGAGCATAGCAAAGGTCAGGG |
| Human _NEAT1_ REVERSE | CTTCCTCCCTTTAACTTATCCAATCAC |
| Human_ROR1_FORWARD | TAATCGGAGAGCAACTTCA |
| Human_ROR1_REVERSE | TGTAGTAATCAGCGGAGTAA |
| Human_GAPDH_FORWARD | TGGCTTTCATCACCTCGTGG |
| Human_GAPDH_REVERSE | GTCTCCTCTGACTTCAACAGCG |
| Human_U6 snRNA_FORWARD | ACCACCCTGTTGCTGTAGCCAA |
| Human_U6 snRNA_REVERSE | CTCGCTTCGGCAGCACATTC |

**MTT ASSAY**

**(a)** **(b)**

**Supplementary Figure 1:** Effect of Aβ_1-42_ on cell viability in SHSY-5Y cells (a). Bar graphs representative of five (n = 5) independent experiments of Cell viability in SHSY-5Y cells, detected by Trypan Blue Exclusion Assay in DMSO treated and Aβ_1-42_ treated cells. (b). Bar graphs representative of 8 (n = 8) independent experiments of Cell viability in SHSY-5Y cells, detected by MTT Assay in DMSO treated cells and Aβ_1-42_ treated cells. Error bars indicate ± SD. The statistical significance level between experimental pairs is indicated (NS, not significant; *,p<0.05; **,p<0.01; ***,p<0.001).

**Supplementary Figure 2: Ratio of neurite length: neurite number in SHSY-5Y cells:** Bar graphs representative of three (n=3) independent experiments measuring neurite length: neurite number in SHSY-5Y cells either over expressing ROR1, treated with Aβ_1-42_ or ROR1+ Aβ_1-42_.

**Supplementary Figure 3:** Quantitation showing the Pan -actin levels in the F and G fractions of untreated cells, GFP transfected cells, ROR1 transfected cells and cells treated with DMSO. In each case, F: G ratio (y-axis) > 1. (n=3)


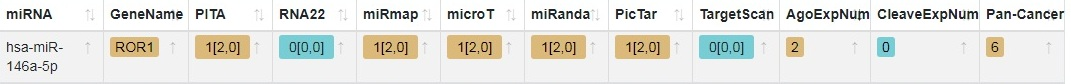


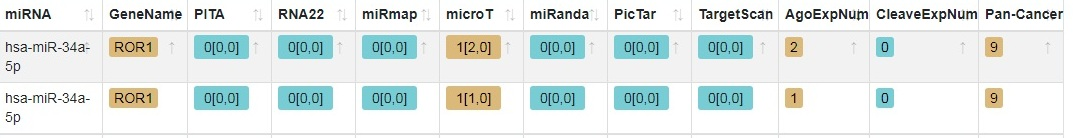


**Supplementary Figure 4: Bioinformatics prediction of interaction of ROR1 with hsa-miR- 146a-5p and hsa-miR- 34a-5p from ENCORI database.**

**Supplementary Figure 5: Endogenous expression levels of hsa-miR- 34a-5p and hsa-miR- 146a-5p:** Bar graphs representative of three (n=3) independent experiments measuring relative endogenous expression levels of hsa-miR- 34a-5p and hsa-miR- 146a-5p in SHSY5Y cells by qRT-PCR. U6snRNAwas used as endogenous control.


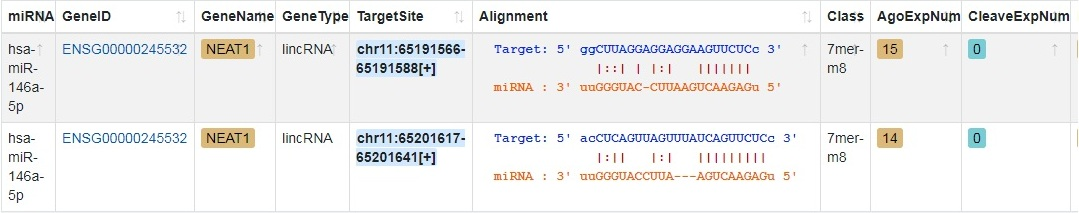


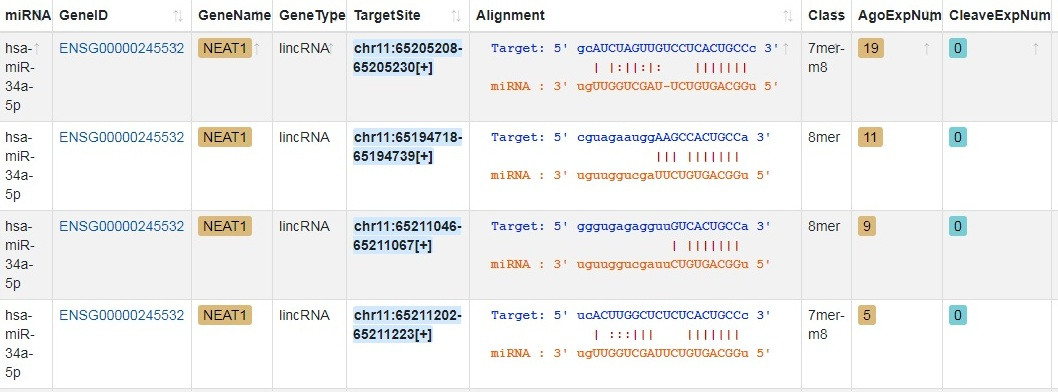


**Supplementary Figure 6: Bioinformatics prediction of interaction of NEAT1 with hsa-miR- 146a-5p and hsa-miR- 34a-5p from ENCORI database.**

**

**Supplementary Figure 7: Decrease in expression of NEAT1 after treatment with siRNA:** Bar graphs representative of three (n=3) independent experiments measuring expression of NEAT1 by qRT-PCR in SHSY5Y cells transfected with NEAT1 siRNA or corresponding negative control siRNA. U6snRNA was used as endogenous control. Fold change was calculated by considering the normalised levels of NEAT1 in negative control siRNA treated cells to be 1. Error bars indicate ± SD. The statistical significance level between experimental pairs is indicated (NS, not significant; *,p<0.05; **,p<0.01; ***,p<0.001).


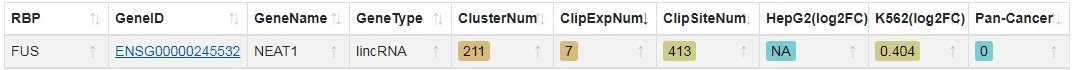


**Supplementary Figure 8: Bioinformatics prediction of interaction of lncRNA NEAT1 with RNA Binding Protein FUS from ENCORI database.**

**
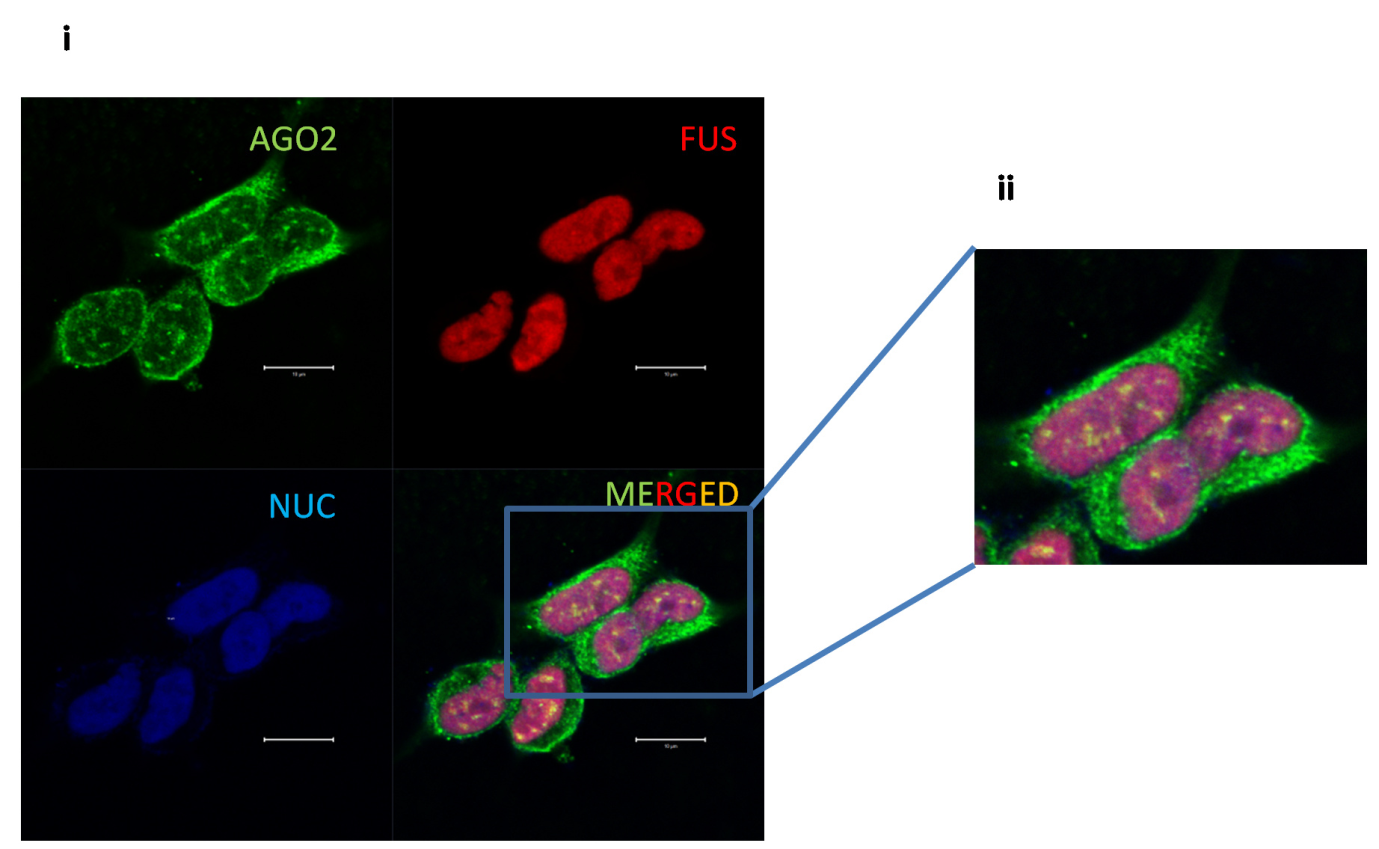
**

**Supplementary Figure 9: Co-localization Analysis - Immunocytochemistry (ICC) assay.** AGO2 with FUS in SHSY5Y cells; panel (i) and panel (ii) – inset- showing specific co- localisation of AGO2 and FUS in intra nuclear clusters. Scale bars, 20μm- For each ICC experiment, images of at least 30 cells (or cell fields) were captured and the experiments were repeated thrice (n=3).


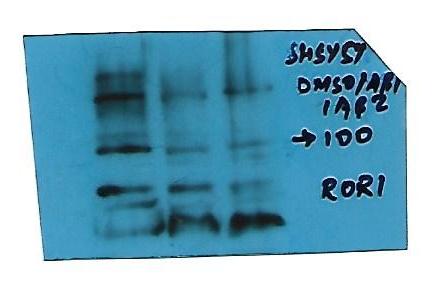
 **
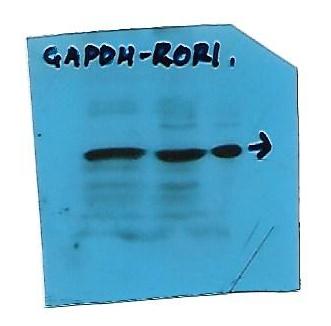
**

**Supplementary Figure 10 a & b: Supplementary Blot for Figure 1 c. ROR1 and GAPDH panel.**

**
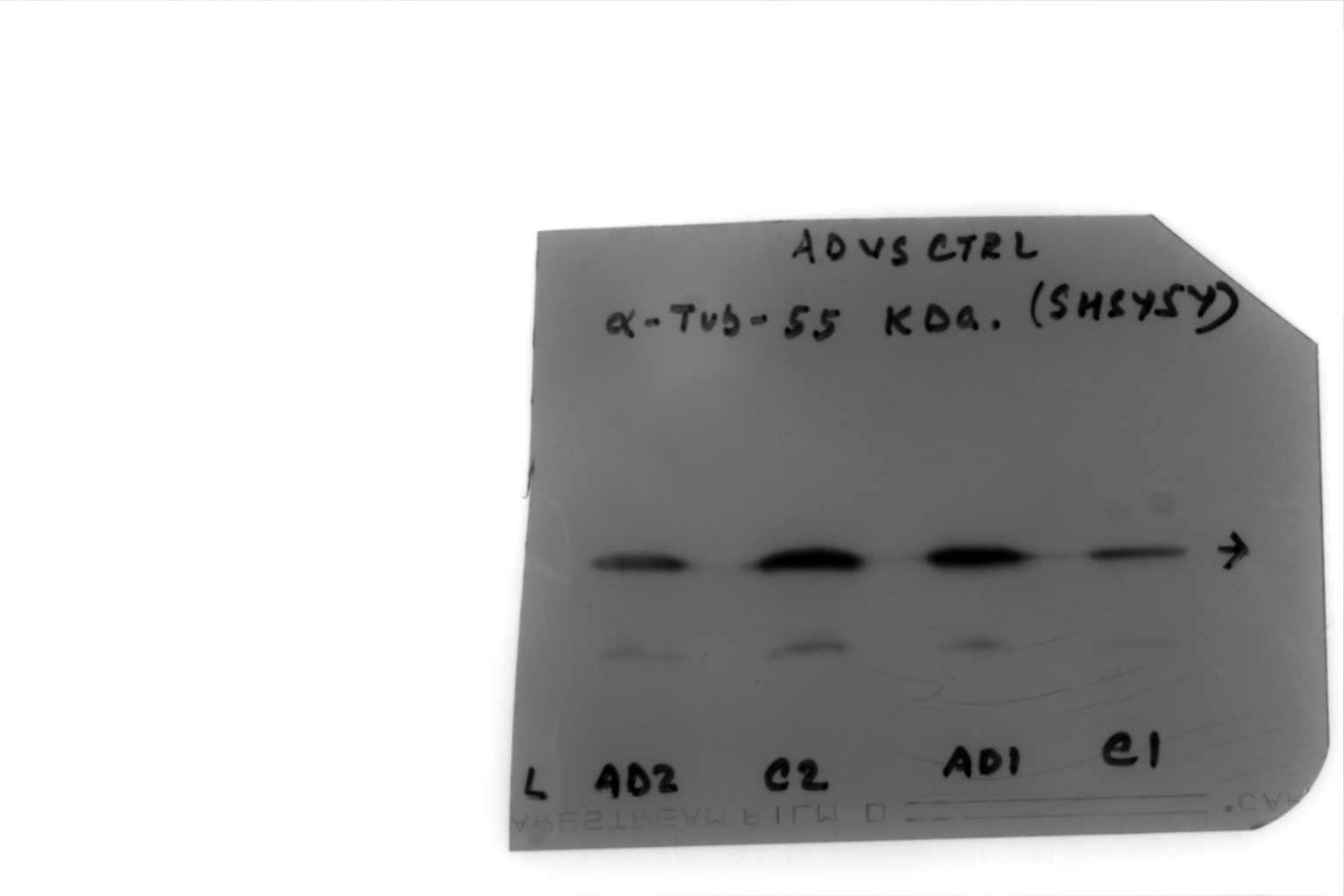
**

**Supplementary Figure 10 c: Supplementary Blot for Figure 1 e. alpha-tubulin panel.**

**
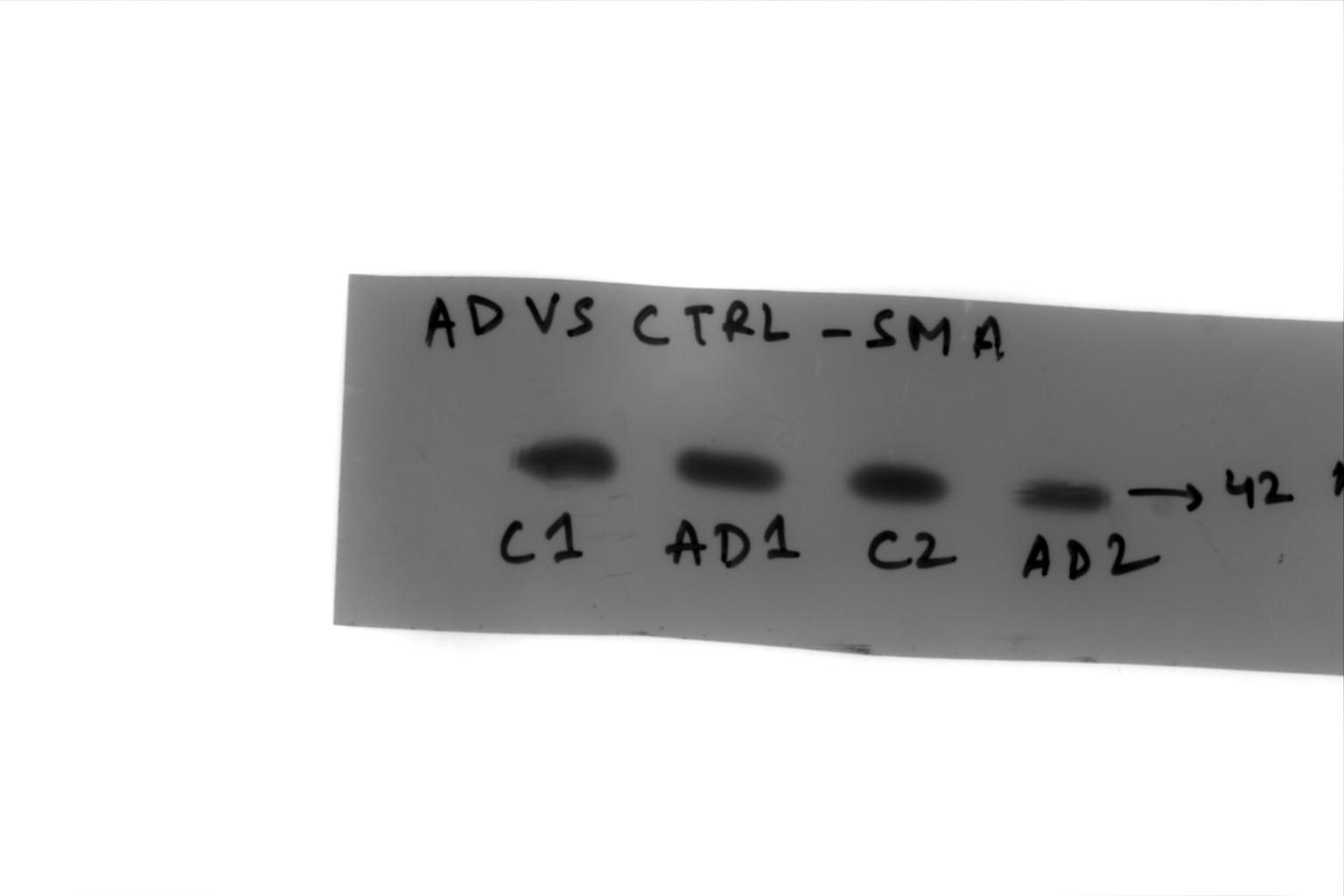
**

**Supplementary Figure 10 d: Supplementary Blot for Figure 1 e. SMA panel.**

**
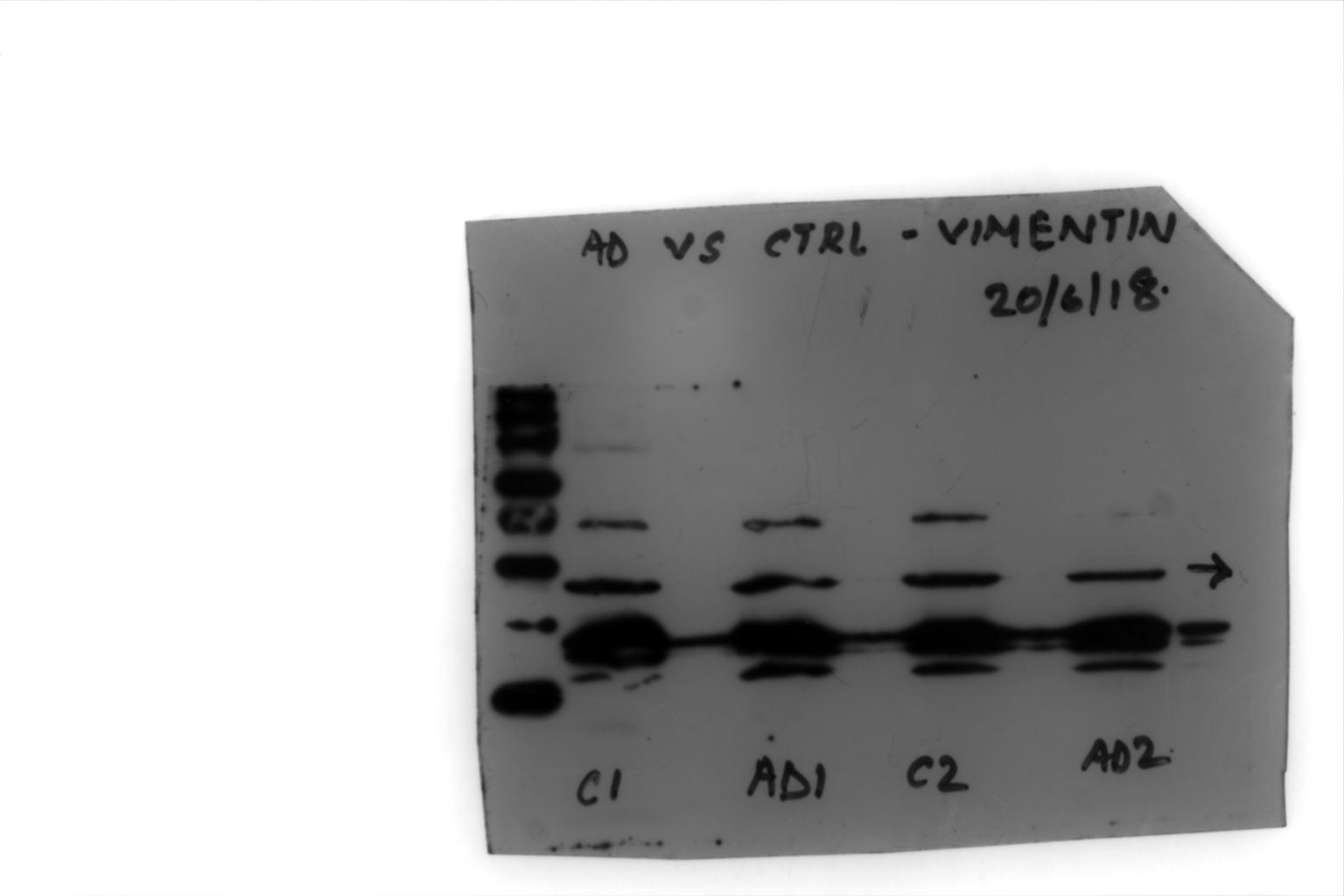
**

**Supplementary Figure 10 e: Supplementary Blot for Figure 1 e. Vimentin panel.**

**
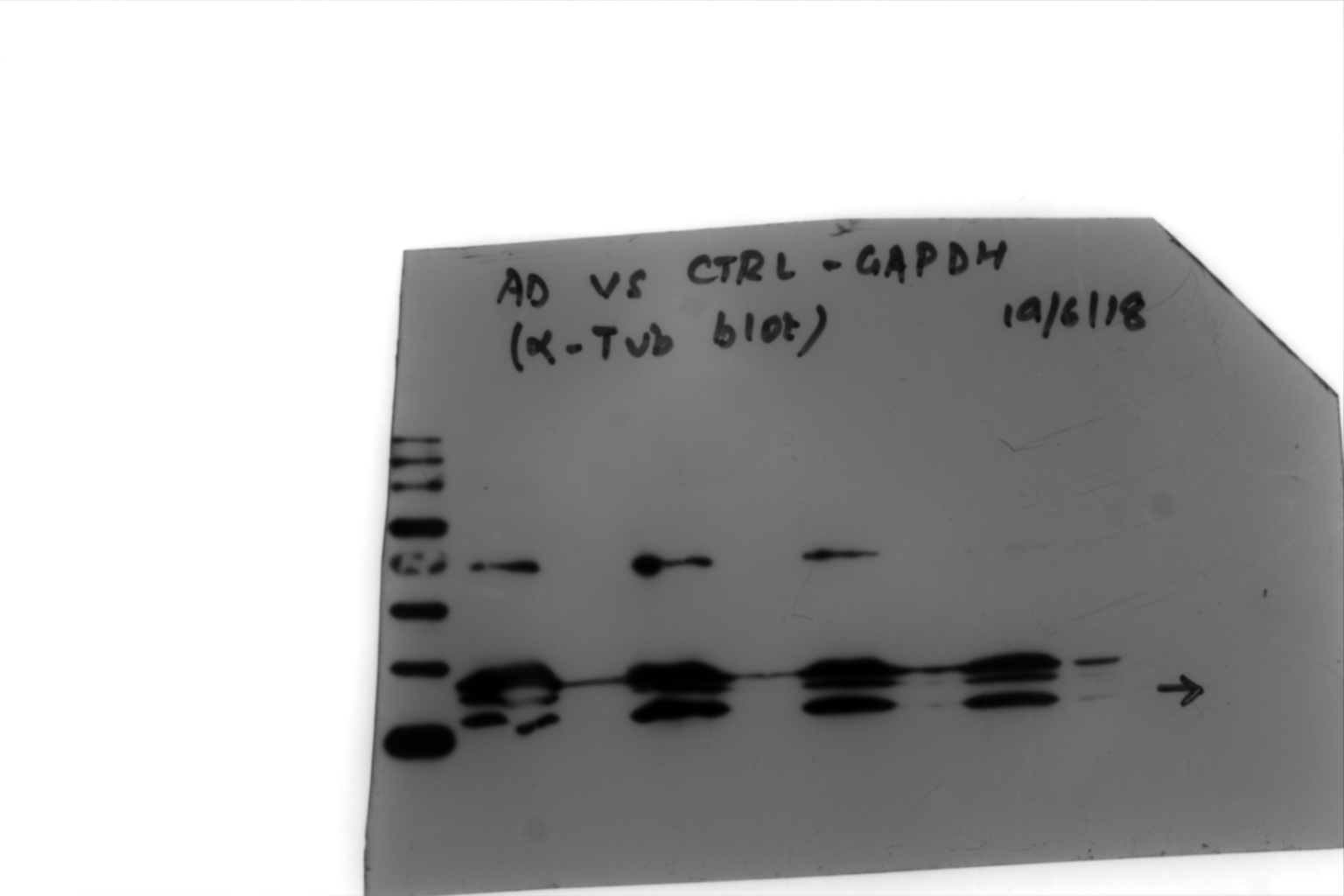
**

**Supplementary Figure 10 f: Supplementary Blot for Figure 1 e. GAPDH panel.**

**
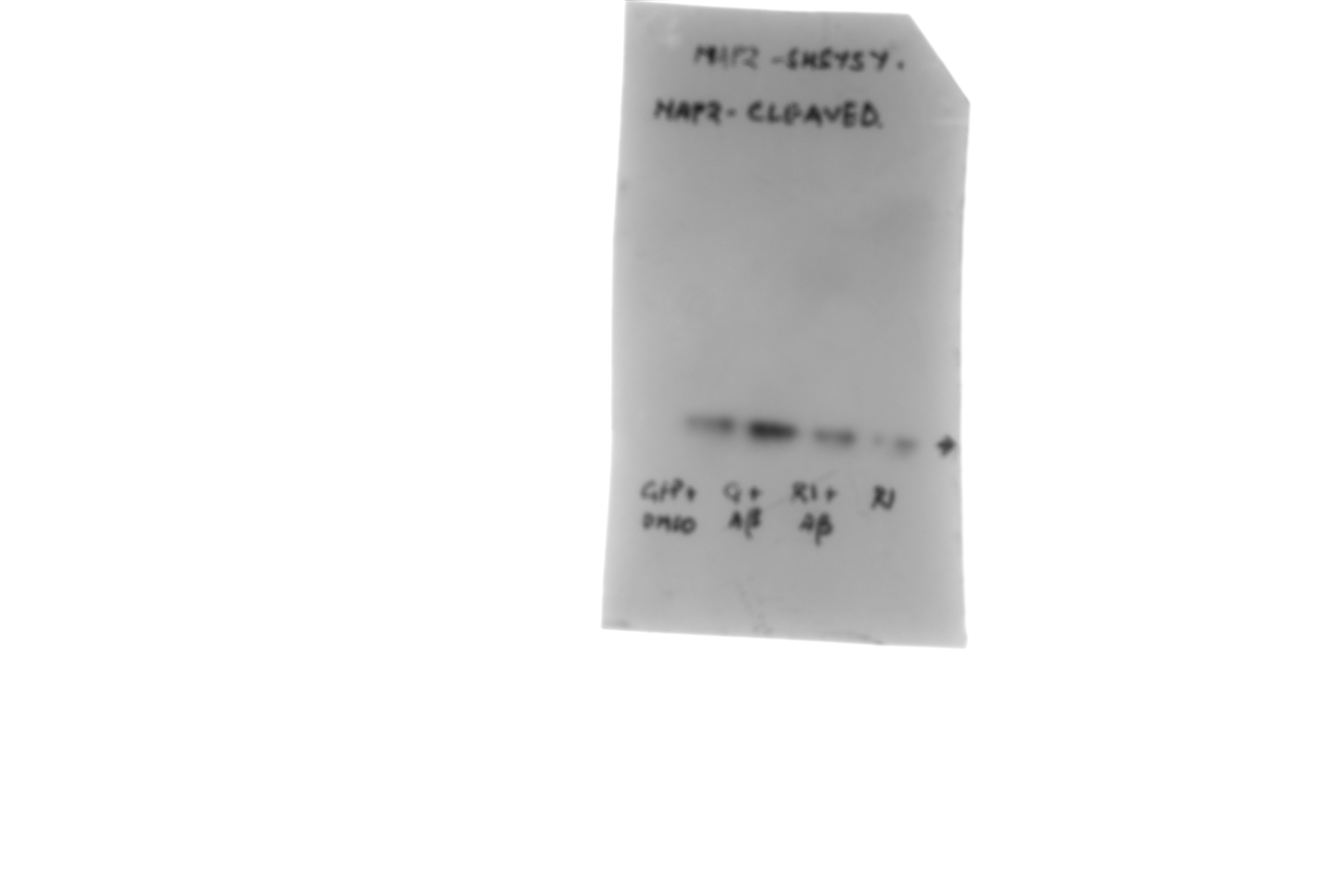
**

**Supplementary Figure 10 g: Supplementary Blot for Figure 2 c. Cleaved MAP2 panel.**

**
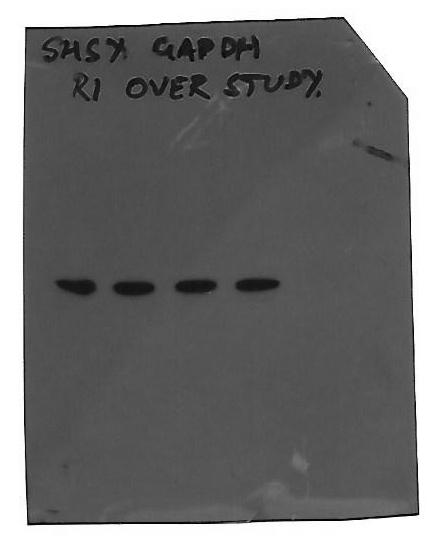
**

**Supplementary Figure 10 h: Supplementary Blot for Figure 2 c. GAPDH panel.**

**
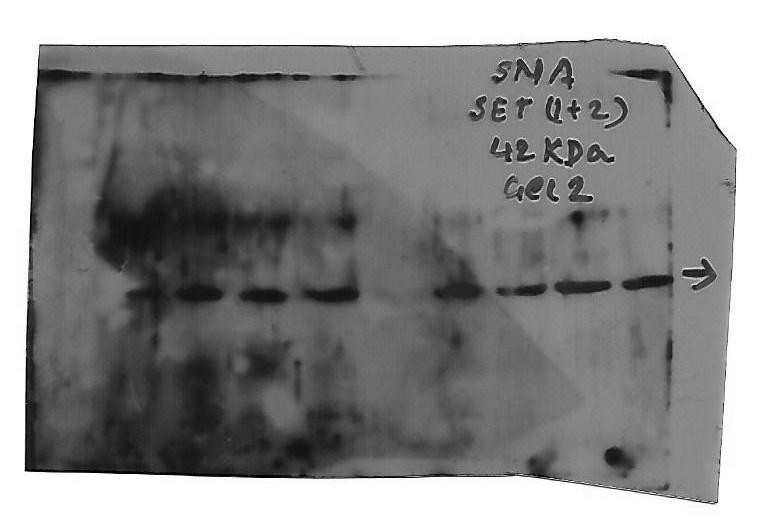
**

**Supplementary Figure 10 i: Supplementary Blot for Figure 2 c. SMA panel.**

**
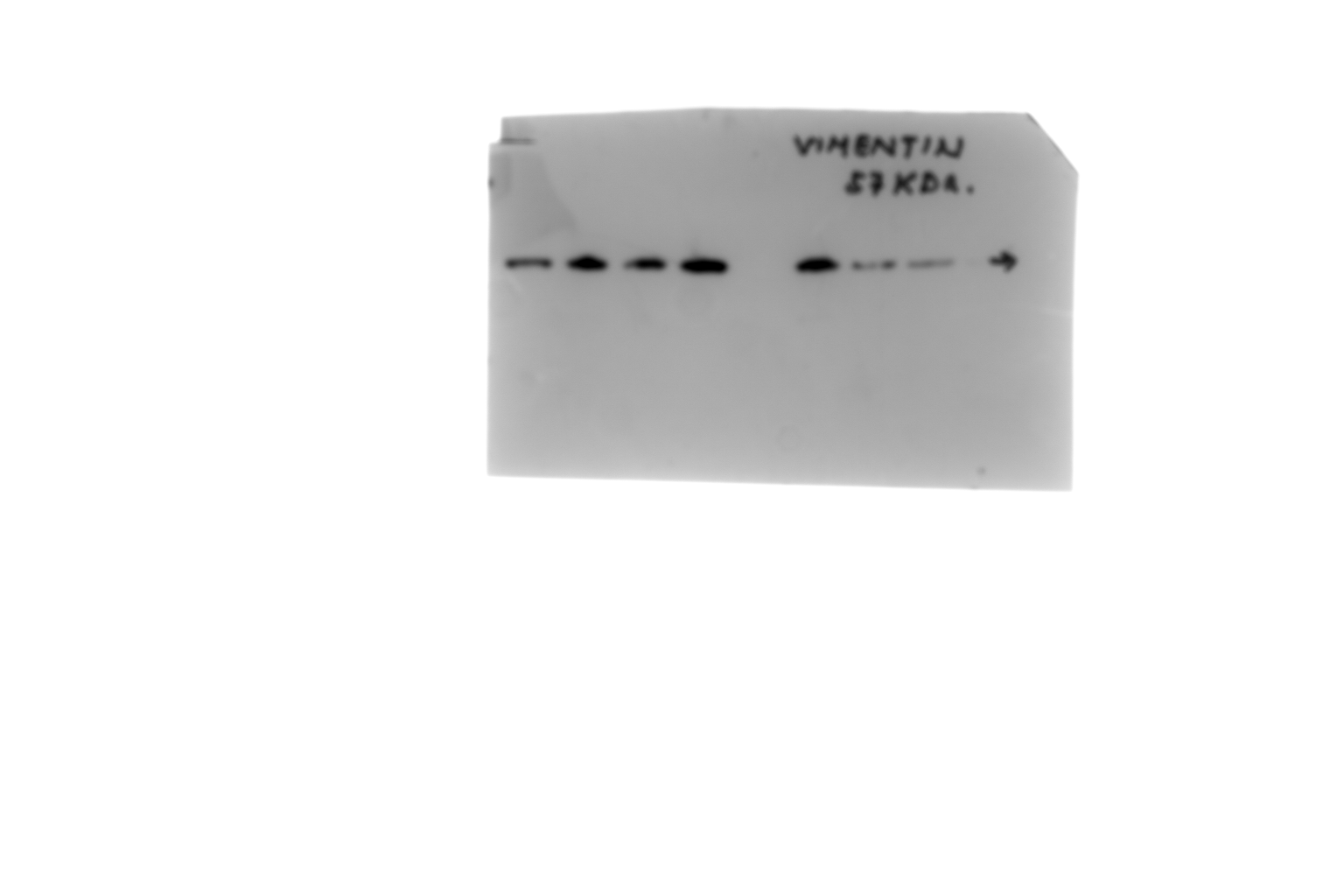
**

**Supplementary Figure 10 j: Supplementary Blot for Figure 2 c. Vimentin panel.**

**
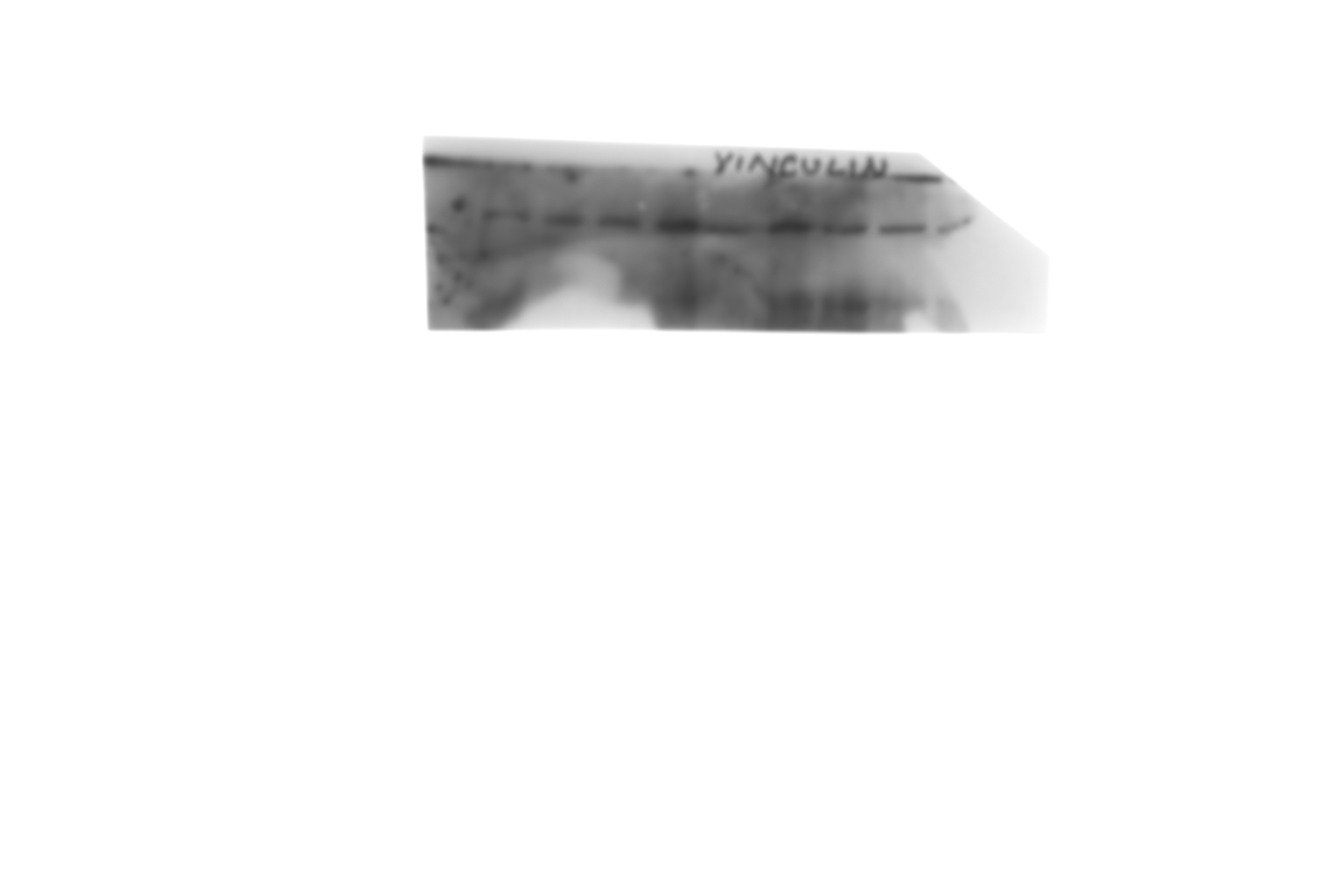
**

**Supplementary Figure 10 k: Supplementary Blot for Figure 2 c. Vinculin panel.**

**
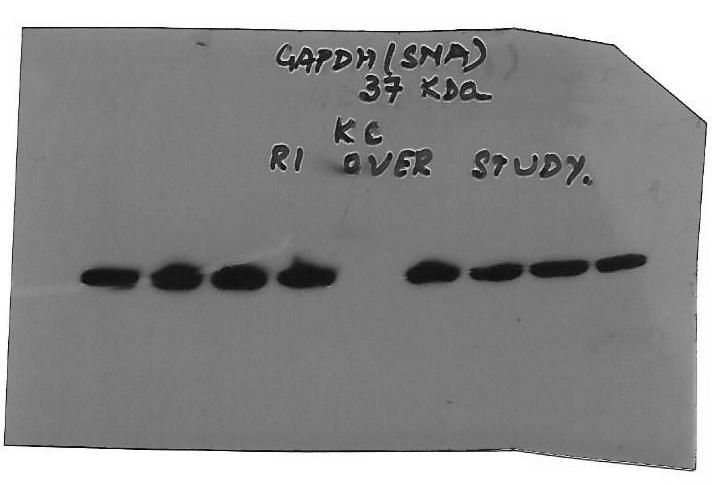
**

**Supplementary Figure 10 l: Supplementary Blot for Figure 2 c. GAPDH panel.**

**
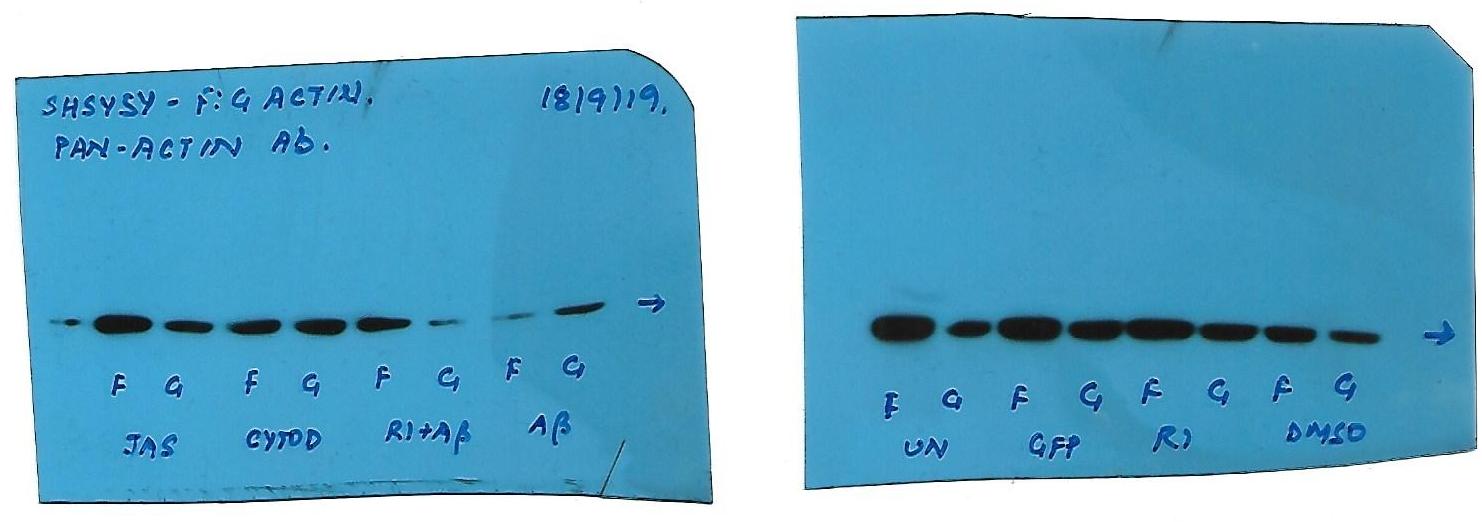
**

**Supplementary Figure 10 m: Supplementary Blot for Figure 3 d. Pan-actin panel.**
